# Revised World Health Organization (WHO)’s causality assessment of adverse events following immunization—a critique

**DOI:** 10.12688/f1000research.13694.2

**Published:** 2018-05-29

**Authors:** Jacob Puliyel, Pathik Naik

**Affiliations:** 1 St Stephen's Hospital, Delhi, 110054, India; 2 Pathik Children Hospital, Surat, 394219, India

**Keywords:** Pentavalent vaccine; quinvaxim; pharmacovigilance; Hill criteria; macrophagicmyofasciitis; periodic safety update reports; Brighton classification; adverse drug reactions; sudden unexpected death; TOKEN study

## Abstract

The World Health Organisation (WHO) has recently revised how adverse events after immunization (AEFI) are classified. Only reactions that have previously been acknowledged in epidemiological studies to be caused by the vaccine are classified as a vaccine-product–related-reaction. Deaths observed during post-marketing surveillance are not considered as ‘consistent with causal association with vaccine’, if there was no statistically significant increase in deaths recorded during the small Phase 3 trials that preceded it. Of course, vaccines  noted to have caused a significant increase in deaths in the control-trials stage would probably not be licensed. After licensure, deaths and all new serious adverse reactions are labelled as ‘coincidental deaths/events’ or ‘unclassifiable’, and the association with vaccine is not acknowledged. The resulting paradox is evident.

The definition of causal association has also been changed. It is now used only if there is ‘no other factor intervening in the processes’. Therefore, if a child with an underlying congenital heart disease (other factor), develops fever and cardiac decompensation after vaccination, the cardiac failure would not be considered causally related to the vaccine. The Global Advisory Committee on Vaccine Safety has documented many deaths in children with pre-existing heart disease after they were administered the pentavalent vaccine. The WHO now advises precautions when vaccinating such children. This has reduced the risk of death. Using the new definition of causal association, this relationship would not be acknowledged and lives would be put at risk. In view of the above, it is necessary that the AEFI manual be revaluated and revised urgently. AEFI reporting is said to be for vaccine safety. Child safety (safety of children) rather than vaccine safety (safety for vaccines) needs to be the emphasis.

## Introduction

One of the earliest countries to introduce the pentavalent vaccine (combined diphtheria, tetanus, pertussis, Hib, and hepatitis B) was Sri Lanka ^1^. A pentavalent vaccine Quinvaxem (Crucell) was introduced in Sri Lanka on January 1, 2008. On 29 April that year the vaccine was withdrawn by the government following five deaths. A World Health Organization (WHO) team of experts investigated the adverse events following immunization (AEFI) and reported the deaths were ‘unlikely’ to be related to vaccination. The full report was not widely available before it was presented to the High Court in Delhi, India ^2^. From the full report it became clear that there was no alternate explanation for three deaths. Thus, they should have been classified as ‘probable / likely’ related to immunization, using the WHO Brighton criteria for classification of AEFI (see Box 1). The experts deleted the categories ‘probable’ and ‘possible’ from the AEFI Classification they used for assessment and then reported that the deaths were ‘unlikely’ related to vaccination. The way the Brighton Classification was altered to enable this misleading classification of the deaths in Sri Lanka was reported in the Indian Journal of Medical Research and the British Medical Journal ^3, 4^.

On 4 May 2013 the Ministry of Health of Vietnam suspended the use of Quinvaxem (Crucell) after it had caused 12 deaths ^5^. The WHO experts investigated the Vietnam deaths. This time they reported, ‘Quinvaxem was pre-qualified by WHO…, no fatal adverse event following immunisation (AEFI) has ever been associated with this vaccine’ ^5^. This is the same brand of pentavalent vaccine that was used in Sri Lanka where WHO experts had previously documented AEFI deaths. It appears that after the Sri Lanka investigation and shortly preceding the Vietnam investigation, the methodology used for AEFI classification was revised. Using the revised AEFI causality assessment, AEFI reported from Sri Lanka could be classified as ‘Not a case of [AEFI]’. Both Sri Lanka and Vietnam were persuaded to reintroduce the Pentavalent vaccine after the WHO report. The new mechanism that allows AEFI to be classified as ‘Not a case of [AEFI]’ will be discussed.

**Box 1.** WHO adverse events following immunization (AEFI): causality assessment Brighton criteria

**Causality Term**	**Assessment Criteria**
Very likely/Certain	A clinical event with a plausible time relationship to vaccine administration and which cannot be explained by concurrent disease or other drugs or chemicals
Probable	A clinical event with a reasonable time relationship to vaccine administration; is unlikely to be attributed to concurrent disease or other drugs or chemicals.
Possible	A clinical event with a reasonable time relationship to vaccine administration, but which could also be explained by concurrent disease or other drugs or chemicals.
Unlikely	A clinical event whose time relationship to vaccine administration makes a causal connection improbable, but which could be plausibly explained by underlying disease or other drugs or chemicals
Unrelated	A clinical event with an incompatible time relationship and which could be explained by underlying disease or other drugs or chemicals
Unclassifiable	A clinical event with insufficient information to permit assessment and identification of the cause

Reference http://www.rho.org/files/rb3/AEFI_Causality_Assessment_WHO_2005.pdf Reproduced with permission.

## Section A

### Historical background of causality assessment: from Hume up to Brighton

The evolution of the logic of causality assessment is fascinating. Eminent philosophers, scientists, legal luminaries, and statisticians have grappled with the issue and a great deal has been written about it. It will be impossible to distil all of that for this write-up, except at the risk of oversimplification. As we are concerned primarily with assigning causality to alleged drug reactions, only some aspects of the debate are germane to this discussion.

Defining cause and effect (X is the cause of Y) has not been easy. According to Hume ^6^, the major features of causation are temporal precedence (X must precede Y), contiguity and regularity of the association of causes and their effects. Confounding, however, is possible by a third factor.

It is known that the consumption of ice cream is higher when there is a spike in the incidence of sunburns. One can conclude wrongly that eating ice cream can cause sunburns. The third factor in this case is hot weather conditions. Both eating ice cream and getting sun burnt are associated with sunny days. Hume avoided the confounding problem by stipulating that X can be considered as cause of Y only if X is sufficient for Y. That is, however, fallacious. Striking a match can light a fire only if there is oxygen. In itself, striking the match is not sufficient. The alternate position could be that X is cause of Y if, and only if, X is necessary for Y ^7^. John Mackie suggested that in nature there could be multiple reasons (causes) for the same outcome ^8^. Thus X may not be necessary for Y but at the same time, X may be sufficient for Y. A building may be set on fire by a spark from a short circuit in the electrical wiring (X) or as the result of an act of arson (Z). Thus neither (X) nor (Z) is necessary for Y, but both (X) and (Z) are sufficient causes for Y. The question then is whether Y would have occurred were it not for the factor X. This is known as the ‘but for’ test. In jurisprudence, it has been acknowledged that where there are multiple causes working simultaneously the ‘but for test is unworkable and the question of causality is whether the putative cause materially contributed to the result ^9^. This has been argued in the case of Graham Dickie V. Flexcon Glenrothes Limited [2009] ScotSC 143 (04 September 2009). Peter M. Willcock and James M. Lepp have discussed ‘Causation in medical negligence cases’ which elaborates on these issues.

In biology, there is a further probabilistic element to causation. If men of the same height and women of the same height were to have children, their children will not all be of the same height. For the same set of observed causal factors, there is probability distribution of possible heights ^7^.

To evaluate causation Bradford Hill ^10^ described 9 guiding principles favouring a causative association: 1) Strength - effect size; 2) Consistency – reproducibility with similar observations at different places by different people;3) Specificity – absence of an alternate explanation; 4) Temporarily with cause always proceeding the effect; 5) Biological gradient demonstrating a dose response gradient; 6) Biological plausibility – although this may be limited by the state of current knowledge; 7) Coherence between epidemiology and laboratory findings; 8) Experimental evidence; and 9) Analogy - looking at the effect of similar factors. These considerations are applicable to alleged vaccine reactions also.

### Adverse drug reactions

Adverse drug reactions (ADRs) can follow after the use of any drug. Careful evaluation is required to distinguish the events that are causally related to the drug from coincidental events. Causality assessment is crucial because the events could be iatrogenic and avoidable. Usually only a few react adversely to drugs on the market, whereas others are unharmed. The attribution of causality for such occasional happenings is particularly complex. Investigations of ADRs put causative association on a probability scale. The causality-assessment system developed by the World Health Organization Collaborating Centre for International Drug Monitoring is called the Uppsala WHO Centre (WHO-UMC) Scale. This is widely used as it offers a simple methodology (see Box 2). In consonance with Hume’s postulates, the first step is to confirm temporal precedence and contiguity. The adverse event must appear after the suspected drug is administered and within a reasonable time-frame. Events where the time-to-drug-intake makes a relationship improbable are classified as ‘unlikely’ to be related. Events within a reasonable time and for which there is no alternate explanation (which cannot be attributed to disease or other drugs) are classified as ‘probable / likely’ related to the drug in question. Drug reaction is classified as ‘possible’ where there is a reasonable time relationship, but for which there are also alternate explanations. In terms of John Mackie’s aphorism, the drug is considered sufficient but not necessary for the effect.

**Box 2.** WHO–UMC causality categories

**Causality term**	**Assessment criteria**
**Certain**	• Event or laboratory test abnormality, with plausible time relationship to drug intake • Cannot be explained by disease or other drugs • Response to withdrawal plausible (pharmacologically, pathologically) • Event definitive pharmacologically or phenomenologically (i.e. an objective and specific medical disorder or a recognised pharmacological phenomenon) • Rechallenge satisfactory, if necessary
**Probable/Likely**	• Event or laboratory test abnormality, with reasonable time relationship to drug intake, Unlikely to be attributed to disease or other drugs • Response to withdrawal clinically reasonable • Rechallenge not required
**Possible**	• Event or laboratory test abnormality, with reasonable time relationship to drug intake • Could also be explained by disease or other drugs • Information on drug withdrawal may be lacking or unclear
**Unlikely**	• Event or laboratory test abnormality, with a time to drug intake that makes a relationship improbable (but not impossible) • Disease or other drugs provide plausible explanations
**Conditional/** **Unclassified**	• Event or laboratory test abnormality • More data for proper assessment needed, or • Additional data under examination
**Unassessable/** **Unclassifiable**	• Report suggesting an adverse reaction • Cannot be judged because information is insufficient or Contradictory • Data cannot be supplemented or verified

Reference The Uppsala Monitoring Center. The use of the WHO-UMC system for standardised case causality assessment. Reproduced with permission of *Uppsala monitoring centre*. Available at https://www.who-umc.org/media/2768/standardised-case-causality-assessment.pdf

To be classified as ‘very likely/certain’ the reaction needs to be an objective and specific medical disorder or a recognized pharmacologic phenomenon, and there must be evidence of dose-related reaction or proof in terms of reappearance of symptoms on rechallenge. If death should occur as ADR, rechallenge is impossible. It is usually difficult to be certain about the causality of fatal ADR and the reaction is often classified as ‘probable/likely’ or ‘possible’.

The difference between certain and probable/likely is simply the acceptable standard of proof. For “certainly,” a high-standard irrefutable proof is called for (falsification of the theory by a single irregular outcome). A single well-documented spontaneous rechallenge is strong evidence of regularity (even though in just one patient). For ‘very likely’, the standard of proof is proof beyond reasonable doubt.

‘Balance of probability’ is the level of proof needed to classify as ‘probable’ or ‘possible’and this is the standard of proof, which is relevant to medicine and for pharmacovigilance. With this level of proof (prima facie true), the 'Precautionary Principle’ must be triggered. This is described later.

### Adverse events following immunization

Vaccines are drugs used as a preventive measure, given to entire cohorts of healthy persons. As they are administered in the absence of any disease, there is very high expectation that they will produce few adverse effects. But there is low tolerance for serious adverse events and deaths. Adverse events following immunization (AEFI) must be monitored more carefully than other drugs. A credible immunization safety evaluation and monitoring system is essential for the success of immunization programmes. The WHO developed the ‘Adverse Events Following Immunization (AEFI): Causality Assessment’ otherwise known as the Brighton Classification. It is very similar to the WHO-UMC causality categories for ADR. Until recently, this was the touch-stone used by WHO experts when AEFI were reported (see Box 1).

One measure of the sensitivity and responsiveness of the WHO-UMC causality categories (which preceded the Brighton classification) is the alacrity with which the rotavirus vaccine RotaShield was withdrawn in 1999 after 12 cases of vaccine-induced intussusceptions were reported. About 1 in 2000 children younger than 2 months of age develops intussusception from other causes. Based on the results of the investigations, the Centre for Disease Control (CDC) estimated that one or two additional cases of intussusception would be caused among each 10,000 infants vaccinated with the RotaShield vaccine. After about 100,000 infants were immunized, the vaccine was withdrawn ^11^. In 2013, the Brighton classification was abandoned and replaced by the revised AEFI classification. The reasoning that prompted the switch away from the Brighton classification has not been stated explicitly in the revised AEFI manual ^12^.

## Section B

### Brighton Abandoned: Revised Causality Assessment

***The Council for International Organizations of Medical Sciences (CIOMS) / WHO: Report on vaccine pharmacovigilance***. In October 2010, after a series of meetings, 40 experts (of whom 19 were industry representatives with possible conflicts of interest) helped rewrite the classification criteria for AEFIs. The document titled ‘Definitions and Application of Terms for Vaccine Pharmacovigilance’ is reported to ‘provide tools for higher excellence of signal detection and investigation of adverse events following immunization’ ^13^.

On page 170 of this 193-page document, under the heading Notes for Guidelines, it is stated in small print: ‘If there is adequate evidence that an event does not meet a case definition, such an event should be rejected and should be reported as ‘Not a case of [AEFI]’. Such evidence is considered adequate, if an exclusion criteria is met, or an investigation reveals a negative finding of a necessary criterion (necessary condition) for diagnosis. Such an event should be rejected and classified as ‘Not a case of [AEFI]”.’ ^13^

The CIOMS/WHO ‘tool for excellence in signal detection’ works by turning a blind eye to AEFI—classifying AEFI as ‘Not a case of [AEFI]’. Not only is the causative association of AEFI to immunization denied, but it is made to appear the AEFI never occurred. Signal detection is no longer possible once AEFIs are removed from the system after being designated as ‘Not a case of [AEFI]’. The story in the *Introduction* above where the WHO asserted in May 2013 that no fatal AEFI has ever been associated with pentavalent vaccine ^5^, suggests the Sri Lanka AEFI deaths ^2^ are now reclassified as ‘Not a case of [AEFI]’ using the CIOMS/WHO tool.

Only reactions that meet case definitions of reactions associated with the vaccine previously are considered. According to the CIOMS / WHO report (page 11), a case definition can be adopted from the standard literature or by the reviewers themselves.

The case definition helps draw on previous epidemiological research and facilitates further research to confirm a causal link. However, excluding causality in relation to an individual event cannot be dependent on that event conforming to a pre-existing case definition. The pejorative use of the term ‘rejected’ (in the statement; ‘Such an event should be rejected and classified as “Not a case of [AEFI]”’), suggests a defensive posture. It has been pointed out previously that reports of AEFIs should be assessed for causality and classified: they are not to be ‘rejected’ ^14^.

### The WHO revised AEFI manual

**Figure 1.**
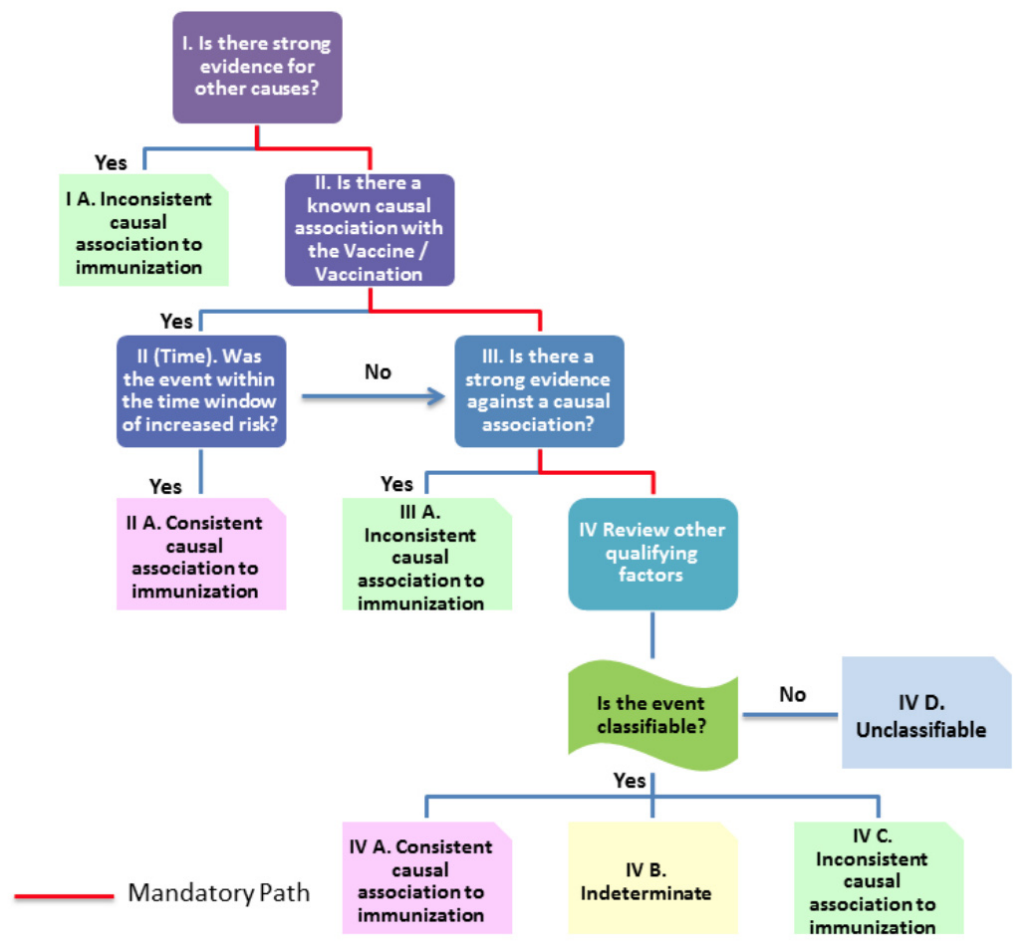
Flow chart demonstrating the revised AEFI classification new algorithm.

In March 2013, the revised WHO ‘User Manual for AEFI’ was published with a new algorithm ^12^. The manual acknowledges that it has adapted definitions and concepts from the CIOMS / WHO report. The new algorithm for AEFI is reproduced in Figure 1.

## Revised AEFI classification: new categories of causality

Only events that occur after vaccine administration are eligible for AEFI causality assessment. This first step is reminiscent of Hume’s dictum regarding precedence and contiguity. In the new scheme, causality is classified in four categories: ‘Consistent causal association to immunization,’ ‘Indeterminate’, ‘inconsistent causal association to immunization’, and ‘Unclassifiable’.

### Consistent causal association to immunization

This is the highest level of causal association in this new classification. It is less definitive than ‘very likely / certain’ in the old scheme. It does not call for irrefutable proof or even proof beyond reasonable doubt. Not even is the balance of probability assessed. In the new scheme, an adverse event can simultaneously be classified as ‘Consistent causal association with immunization’ and ‘Inconsistent causal association with immunization’. On page 36 of the revised manual for AEFI ^12^ is the example of acute flaccid paralysis in a child after oral polio vaccine, who had had a fever 1 month prior to onset of paralysis. The stool culture showed vaccine strain polio virus. It was classified as ‘Consistent causal association with immunization’ as it is a known reaction after polio vaccination and the paralysis happened within time window of increased risk. It was also classified as ‘Inconsistent causal association with immunization’ because the fever, 1 month prior to paralysis had not been investigated completely. This ambiguity, which admits diametrically opposite conclusion simultaneously, is a hallmark of the new scheme.

It is suggested in the revised AEFI manual that before the question ‘Did the vaccine given to a particular individual cause the particular event reported?’ (the question of ‘Did it?’) is answered, one has to answer the question ‘Can the given vaccine cause a particular adverse event?’ (Can it?). The inference is that only if there is evidence at the population level that the vaccine can cause the adverse event, is the reaction classified as ‘Consistent with causal association with immunization’.

This inference is flawed on two grounds. On the one hand, it denies all new associations seen in Phase 4 trials. On the other, if it is a known adverse reaction, causal association is accepted even where the events could have happened by coincidence. Just because intussusceptions are acknowledged as an adverse event following rotavirus vaccination, it does not follow that all intussusceptions in the critical window of increased susceptibility are necessarily caused by it. The residual uncertainty at this highest level of causal association robs it of value in addressing the problem of AEFI caused by vaccines.

### Inconsistent causal association to immunization

At the bottom of the new causality classification hierarchy is ‘Inconsistent causal association to immunization’. This group can include reactions for which there is no alternate explanation (and which would have been classified in the ‘Probable’ category previously). They would fall in the group ‘Inconsistent causal association with vaccination’ merely because causal association with immunization has not been documented in prior epidemiological studies. Into the same group are placed reactions that would have been considered ‘Unlikely’ to be associated, and those that would have been classified as ‘Unrelated’. The use of the same category ‘Inconsistent causal association to immunization’ for such a wide variety of clinical situations merely obfuscates the issues. In the revised scheme, this term is used to suggest that there is no relation between the AEFI and immunization. No matter how frequently the reaction categorized as ‘Inconsistent with causal association’ occurs, it would not be investigated as a new signal of a causal association.

### Indeterminate

Classification in the ‘Indeterminate’ group is reserved for reactions that could have been caused by immunization, but for which causal association has not been documented previously. It is projected that information on AEFI that are classified as indeterminate will be pooled and analysed in order to understand if the AEFI represents a new signal of an unrecognized event. The scheme is however loaded such that literally no AEFI are categorized into this group. How this is accomplished is discussed later on.

### Unclassifiable

Clinical events with insufficient information to permit assessment and identification of cause are put in the ‘Unclassifiable’ group.

## Revised AEFI classification: the new algorithm

Just as the final categories of causality association are vague, overlapping, and not clearly differentiated, the algorithm used to make a decision on causality ^12^ does not appear to be logical or well thought through.

The algorithm is shown in Figure 1.

## Causality assessment algorithm

Four sets of questions need to be answered in sequence:

1. Is there strong evidence of other causes?
2. Is there known causal association with the vaccine or vaccination and if so, whether the event was within the time window of increased risk?
3. If there is no causal association known or if it is not within the time window of increased risk: Is there strong evidence against a causal association?
4. If there is no such strong evidence against causal association, the next step is to look at other qualifying factors for classification:
  a. Could it happen independently of vaccination (background rate)?
  b. Could the event be manifestation of another health condition?
  c. Did a comparable event occur after a previous dose of a similar vaccine?
  d. Was there exposure to a potential risk factor or toxin prior to the event?
  e. Was there acute illness prior to the event?
  f. Did the event occur in the past independently of vaccination?
  g. Was the patient taking any medication prior to vaccination?
  h. Is there biological plausibility?

### Step 1

The first step in the revised algorithm is to look for strong evidence for other causes. If there is an alternate explanation, the AEFI is classified as ‘Inconsistent with causal association to immunization’. John Mackie has noted that in nature there could be multiple reasons (causes) for the same outcome, and if two possible causes exist simultaneously either of them could be the causative factor ^8^. It is to be noted that with the WHO-UMC classification of ADR and the old WHO/Brighton Classification of AEFI, even if an alternate explanation is available, a causative association with drug or vaccine is still considered ‘Possible’. Moreover, the two causes could be working synergistically. An example of this is where genetic and other individual susceptibility factors make one susceptible to developing an AEFI ^15, 16^. In the new algorithm, if there is an alternate explanation for the AEFI, or another factor is involved, causative association with vaccine is rejected ^12, 14^.

### Step 2

The COIMS / WHO Report on pharmacovigilance is used at this level ^13^. AEFI-specific case definitions for some reactions have been developed. In instances where specific case definitions and criteria are not available for a particular AEFI, it is permissible to improvise using case definitions adopted from ‘standard medical literature, or national guidelines or they may be adopted locally by the reviewers’ (page 11 CIOMS / WHO report). AEFI that meet case definitions and which occur within the time window of increased risk are classified as ‘consistent causal association to immunization’.

The acceptable time window for each adverse event is different. The macrophagic myofasciitis affected patients usually are middle-aged adults presenting diffuse arthromyalgias, chronic fatigue, and marked cognitive deficits, fatigue, or depression due to long-term persistence of aluminium hydroxide within macrophages at the site of previous immunization ^17^. However, AEFI surveillance seldom extents for so long.

### Step 3

Theoretically, reactions that are not known to have a causal association or those that are not in the time window of increased risk can move to Step 3. At this stage, an enquiry is made whether there is strong evidence against causal association. Proving of a negative is notoriously difficult as it is impossible to affirm that in every circumstance, an irregular outcome is impossible. The example provided in the manual relates to MMR and autism.

It is reported that the Global Advisory Committee on Vaccine Safety (GACVS) and Council for International Organizations of Medical Sciences (IOM committee) have concluded that no evidence exists of a causal association between MMR vaccine and autistic disorders. Such AEFI must be classified as ‘inconsistent with causal association to immunization’ according to the new algorithm.

After publication of this AEFI user’s manual, the conclusion about MMR and autism have become disputed again (see Box 3). This shifting evidence calls into question the usefulness of introducing this step in the algorithm of AEFI.

**Box 3. MMR and autism risk in African American children.**

In 2004 the CDC published research demonstrating that there was no link between the vaccinated children’s risk of a subsequent diagnosis of autism and the age at which the child is vaccinated with MMR ^a^. It has now been revealed through the testimony of one of the authors Dr. W. W. Thompson who turned whistle blower, that the risk of autism among African American children vaccinated before the age of two years was 340% that of those vaccinated later. However this data was deliberately removed from the analysis to arrive at the CDC’s proclaimed conclusion. CNN published the story of the CDC whistle-blower ^b^, and Thomson was granted whistleblower immunity by the Obama administration. ^c^

**References:**

a. DeStefano F, Bhasin TK, Thompson WW, Yeargin-Allsopp M, Boyle C. Age at first measles-mumps-rubella vaccination in children with autism and school-matched control subjects: a population-based study in metropolitan Atlanta. Pediatrics. 2004;113:259–66. doi: 10.1542/peds.113.2.259
b. Goldschmidt D. Journal questions validity of autism and vaccine study [Internet]. CNN.com. 2014 Aug 28 [cited 2014 Sep 29]. Available from: http://edition.cnn.com/2014/08/27/health/irpt-cdc-autism-vaccine-study
c. http://dailycaller.com/2015/02/03/obama-admin-grants-immunity-to-cdc-scientist-that-fudged-vaccine-rep4rt-whistleblower-plans-to-testify-before-congress/

### Step 4

Assuming that no such ‘strong evidence against a causal association’ exists, reactions that are not known to have a causal association with the vaccine, can go to Step 4. It is from here that reactions may be classified as indeterminate allowing it to be evaluated in future as a new signal.

The question at this point is whether it is ‘classifiable — meaning whether all the tests needed have been performed to allow it to be classified under the CIOMS / WHO definitions. This is the second time these definitions are invoked during the AEFI evaluation.

If some investigations are not done or not available, the AEFI is labelled as ‘Unclassifiable’ (or classified as ‘Inconsistent with causal association to immunization’ like how flaccid paralysis following OPV was classified, because investigations during an illness 1 month prior to paralysis were not available — see Appendix 3, page 36 of the AEFI manual ^12^ for this example).

If all the required investigations had been done and they met case definition criteria, they would have been classified as ‘consistent causal association to immunization’ at Step 2 and would not have come to Step 4.

The third possibility is that all the investigations had been done so it is classifiable but it did not meet case definitions. The CIOMS / WHO dictum is applied here: ‘if there is adequate evidence that an event does not meet a case definition, such an event should be rejected and should be reported as “Not a case of [AEFI]”. (See CIOMS / WHO Definitions and Application of Terms for Vaccine Pharmacovigilance, page 170 ^13^). It removes any chance that AEFI that has not been recognized as causatively associated with immunization in previous epidemiological studies will be included in the ‘Indeterminate’ group and evaluated as a new signal. Thus there seems to be only two options at step 4 : - either the reaction is classified as ‘Unclassifiable‘ or it is categorized as ‘Inconsistent causal association to immunization’. Categorization as ‘Indeterminate’ or ‘Consistent causal association to immunization’ are logically impossible given the riders mentioned above.

The exercise does not end there. Other qualifying factors are also enquired into at Step 4. It is recommended that alternate explanations in terms of background rate, other health conditions, exposure to a potential risk factor or toxin, acute illness, and other medication are again enquired into. Many of these ‘other qualifying factors’, like prior illness and concurrent drug use would presumably have been eliminated at Step 1 when looking for evidence for other causes. This enquiry is repeated again at Step 4 quite unnecessarily. Box 4 illustrates how, in spite of there being epidemiological evidence (the TOKEN Study) that pentavalent vaccine can cause sudden unexpected death, the numerous deaths (as discussed in the introduction) are not acknowledged as caused by the vaccine, and the WHO expert report denies that deaths were ever reported as AEFI. The causality assessment of 132 serious AEFI cases uploaded on the website of the Ministry of Health and Family Welfare in India illustrates the consequence of deploying this new classification. 54 of these babies died, whereas 78 survived. The causality assessment found 50% of those who survived had reactions to vaccination but not even one death was classified as vaccine-related. Nearly all the deaths (96%) were simply classified as unclassifiable or coincidental, presumably because death has not previously been acknowledged as an adverse event caused by this vaccine ^18^. Children admitted to hospital after vaccination with intractable convulsions, could be classified as having a vaccine-product related reaction, but if they died, the deaths would be classified as ‘coincidental deaths’.

**Box 4. Sudden unexpected deaths (SUD) after pentavalent vaccine and the TOKEN Study.**

With regard to AEFI a cluster of cases is defined as two or more cases of the same adverse event related in time or place or to the vaccine administered ^a^. Pentavalent vaccine has caused numerous deaths in Asia but it is yet to be considered a new signal ^b– f^.

After the AEFI algorithm was revised, the deaths are now classified as 'Not a case of [AEFI]' on the grounds that deaths have not been reported as AEFIs in epidemiological studies involving the vaccine. However, the TOKEN Study contradicts this assertion ^g^.

The TOKEN Study was done specifically to assess a possible causal relationship between vaccination and unexplained sudden unexpected death (SUD) of children between their 2nd and 24th month of life. vonKries had previously found a statistically significantly increased standardized mortality ratio (SMR) within two days after vaccination with one (Hexavac®) of the two licensed hexavalent vaccines and the TOKEN study was done to confirm or refute the association ^g^. The study was sponsored and supported by the Paul-Ehrlich-Institute (PEI) and the Federal Ministry of Health (Bundesministeriumfür Gesundheit).

A self-controlled case series (SCCS) was examined to look for a temporal association of vaccination to SUD. Parents were invited to participate in the study if their child had died of SUD. 37.6% of the eligible parents participated. The researchers found that parents were twice as likely to participate if their child had died within one week of vaccination. They used an inverse probability weighted analysis to compensate for this bias. The authors note that this was helpful to overcome the selection bias in infants under 9 months, but even so, the results are still likely to overestimate the risk of SUD in older children.

The weighted SCCS analysis, relative risk of SUD after pentavalent vaccination (first and second year of life) looking at risk period 0–3 days after vaccination versus control period 4–28/183 days showed RR of 8.11 (p= 0.006, 95% CI=1.81-36.24; Table 41 in the TOKEN Report). The weighted SCCS analysis, relative risk of SUD after hexa- or pentavalent vaccination (1st and 2nd year of life) looking at risk period 0–3 days versus control period 4–28/183 days was RR.2.19 (p= 0.031, 95% CI=1.08-4.45; Table 36 in the TOKEN Report)

It is clear from the above that there is reasonable evidence in epidemiological studies that SUDS can occur as AEFI following use of the pentavalent vaccine and the deaths following the use of this vaccine should not be a priori classified as ‘Not a case of [AEFI]’.

**References**

a. http://vaccine-safety-training.org/detection-and-reporting.html
b. Lone Z, Puliyel J. Introducing pentavalent vaccine in the EPI in India: A counsel for caution. Indian J Med Res 2010; 132,: pp 1-3
c. Puliyel J. AEFI and the pentavalent vaccine: looking for a composite picture. Indian J Med Ethics. 2013;10:142-6.
d. No authors listed. Global Advisory Committee on VaccineSafety,12–13 June2013. Wkly Epidemiol Rec. 2013;88:301-12. Available at http://www.who.int/vaccine_safety/committee/reports/Jun_2013/en/ Accessed on 1/11/15
e. Sreedhar S, Antony A, Poulose N. Study on the effectiveness and impact of pentavalent vaccination program in India and other south Asian countries. Hum Vaccin Immunother. 2014;10:2062-5.
f. Martin Schlaud M, Poethko-Müller C, Kuhnert R, Hecker H Robert Koch Institute. Study on deaths in young children (2nd to 24th month of life) (TOKEN Study) http://www.rki.de/DE/Content/Gesundheitsmonitoring/Studien/Weitere_Studien/TOKEN_Studie/Studyreport.pdf?__blob=publicationFile
g. vonKries R, Toschke AM, Strassburger K, Kundi M, Kalies H, Nennstiel U, Jorch G, Rosenbauer J, Giani G. Sudden and unexpected deaths after the administration of hexavalent vaccines (diphtheria, tetanus, pertussis, poliomyelitis, hepatitis B, Haemophiliusinfluenzae type b): is there a signal?. Eur J Pediatr. 2005;164:61-9.

## Other subtle changes in the definition of terms

### ‘Causal association’ redefined

The term causal association now means ‘a cause-and-effect relationship between causative factor and a disease with no factor intervening in the processes’. This is a major step backward for patient safety. The old scheme recognized, for example, that an elderly person with chronic cardiac failure might develop symptoms of cardiac decompensation after influenza vaccination due to a vaccine-caused elevation in temperature or stress from a local reaction at the site of vaccination. The vaccine is therefore considered to have contributed to cardiac failure in this specific situation ^19^. Under the new scheme, this outcome would not be considered as causally related to the vaccine. The question of whether the death would have occurred at that time, had it not been provoked by immunization, would not be acknowledged. Without this recognition, many elderly persons may be exposed to this risk of death unnecessarily when using this vaccine. If the vaccination of an infant was reported to have been followed by sudden death but the child was malnourished or otherwise unwell it does not mean that causality assessment should conclude no cause and effect relationship between the vaccine and the death. There is no scope in this definition to consider interacting causalities ^14, 15^. The Global Advisory Committee on Vaccine Safety has documented many deaths in children with pre-existing heart disease after they were administered the pentavalent vaccine. The WHO now advises precautions when vaccinating such children and this has reduced the risk of death ^1^. Using the new definition of causal association, this relationship would not be acknowledged and lives would be put at risk.

According to Collet and colleagues, it is possible that some individuals experience greater immunogenic response to vaccines compared to the general population and therefore, understanding genetically determined predispositions to developing AEFIs is important ^19^. However, these considerations will not be accounted for, in the new CIOMS /WHO causality assessment scheme. The contribution of vaccine in precipitating encephalopathy in patients who are susceptible on account of genetic factors will also not be considered ^15^. Berkovic has used genetic analyses to identify *de novo* mutations in the sodium channel gene SCNIA in patients with alleged vaccine-induced encephalopathy ^16^. Unwisely, in all these cases the contribution of the vaccine in precipitating the encephalopathy will be ignored.

It is a pity that after all these years, the authors should fall for the Hume fallacy that causality can be claimed only if X is sufficient in itself for Y. The fact that the immunization could have ‘materially contributed’ to the adverse events is ignored.

## Biological plausibility

Biological plausibility is one of the Bradford Hill ‘guiding principles’ that favor causative association ^10^. However, this is limited by the state of current knowledge and it should not be used in itself to deny causative association. For example it is now acknowledged that high-titer measles vaccine is associated with excess female mortality ^20^. The recognition of this association was delayed because of the absence of a biologically plausible explanation. WHO experts now acknowledge that vaccines have non-specific effects which up-regulate or down-regulate both the innate and the adaptive immune system and this can influence child survival ^21^.

The association of intussusception with rotavirus vaccination was also accepted at a time when a biologically plausible explanation was not available ^11^ (See Box 5). Vaccine can therefore have both non-specific beneficial effects and also unexpected deleterious effects which should not be disregarded simply because a ready explanation for the same is not available at the time when it is first noticed.

**Box 5. Indian Rotavirus vaccine trials**

**The prequalification of Rotavac without safety data**

RotaShield was withdrawn as it caused 1 excess case of intussusception per 10,000 children given the vaccine ^11^.

However, a new rotavirus vaccine Rotavac (Bharat Biotec ) was licensed in India after a trial in 3 centres where the vaccine was administered to a total of 4500 children (a sample size too small to show up a rare event that occurs 1 in 10,000) ^a, b^. In spite of this small sample it appears intussusceptions were so common with this vaccine ^c^ in one of the centres (Vellore), it was significantly higher than controls. The trial doctors refused to provide this segregated data in spite of repeated requests ^d^. The government promised to monitor safety in a post marketing surveillance. However, the participants in this trial were not explained the risk seen in the RCT (as is mandatory for ethical clinical trials) and surveillance was for a limited window period of a few weeks after vaccination, whereas the adverse events noticed in the RCT were outside that window period. In remote parts of this country where the vaccine is deployed, in the absence of pediatric surgeons and radiologists, deaths from intussusception are likely to be misclassified as deaths from dysentery.

Even before the data of this post marketing surveillance is available, the WHO recently prequalified the vaccine to be used internationally ^e^.

Clinical trials of other rotavirus vaccines that reduce rotavirus diarrhoea but does not reduce overall incidence of diarrhoea ^f^ and another vaccine that increases the overall incidence of diarrhoea ^g^ instead of decreasing it, have been published.

**References**

a. Bhandari N, Rongsen-Chandola T, Bavdekar A, John J, Antony K, Taneja S, Goyal N, Kawade A, Kang G, Rathore SS, Juvekar S, Muliyil J, Arya A, Shaikh H, Abraham V, Vrati S, Proschan M, Kohberger R, Thiry G, Glass R, Greenberg HB, Curlin G, Mohan K, Harshavardhan GV, Prasad S, Rao TS, Boslego J, Bhan MK; India Rotavirus Vaccine Group. Efficacy of a monovalent human-bovine (116E) rotavirus vaccine in Indian children in the second year of life. *Vaccine*. 2014 Aug 11;32 Suppl 1:A110-6. doi: 10.1016/j.vaccine.2014.04.079. https://www.ncbi.nlm.nih.gov/pubmed/25091663
b. John J, Kawade A, Rongsen-Chandola T, Bavdekar A, Bhandari N, Taneja S, *et al*. Active surveillance for intussusception in a phase III efficacy trial of an oral mono- valent rotavirus vaccine in India. *Vaccine*. 2014;32(August (Suppl. 1)):A104–9/ http://dx.doi.org/10.1016/j.vaccine.2014.03.036.
c. Bajaj J, Puliyel JM. Intussusception risk with 116E rotavirus vaccine in Vellore, South India. *Vaccine*. 2016 Jan 20;34(4):403. doi: 10.1016/j.vaccine.2015.03.007. Epub 2015 Mar 21. https://www.ncbi.nlm.nih.gov/pubmed/25800733
d. *Puliyel JM.* 116 E Rotavirus Vellore Study: Request for Vaccine Safety Data. Response to Indian rotavirus vaccine concern over intussusception is unfounded, say researchers. http://www.bmj.com/content/350/bmj.h2867/rapid-responses
e. https://www.businesswire.com/news/home/20180124005715/en/World-Health-Organization-Grants-Prequalification-Bharat-Biotech%E2%80%99s
f. Kulkarni PS, Desai S, Tewari T, *et al*. A randomized Phase III clinical trial to assess the efficacy of a bovine-human reassortant pentavalent rotavirus vaccine in Indian infants. *Vaccine*. 2017. https://www.ncbi.nlm.nih.gov/pubmed/28967523
g. Kaur J, Puliyel J. Heat-stable oral rotavirus vaccine. *N Engl J Med*. 2017; 377:302 https://www.ncbi.nlm.nih.gov/pubmed/28328346

### Biological plausibility redefined

The meaning of the term biological plausibility has itself been redefined in the Revised AEFI manual. The manual specifies that biological plausibility can only be invoked when laboratory findings or symptom or sign are similar or consistent with natural history and pathophysiology of the infection or antigen. Other biologically plausible explanations (demonstrating there is a mechanism and capacity to lead from the cause to the effect) ^7^, do not qualify. The four approaches to ascertaining causality described by Brady include detection of neo-Humean regularity, examining the counterfactual, experimental manipulation and examining mechanisms and capacities ^7^. The new AEFI recognizes only the experimental approach to the exclusion of other valid approaches and, as a result, can fail to detect causality in a number of cases resulting in harm.

## Chronic fatigue syndrome and the HPV vaccine trial

The above discussion has assumed that adverse events that are reported in the original prelicensure randomised control trials, would be classified as adverse events known to be associated with the vaccine.

Slate investigated randomised trials of human papillomavirus (HPV) vaccines and found that potential side effects were collected for only two weeks in the year-long study. After 2 weeks, individual trial investigators decided, on personal judgment, whether to report medical problems as adverse events. Often they listed new problems as ‘new medical history’. Myalgic encephalomyelitis, otherwise known as chronic fatigue syndrome (CFS), is a condition characterized by long-term fatigue that limits a person’s ability to carry out ordinary daily activities. Participants in the HPV trial reported to Slate that these debilitating symptoms of theirs were not even registered as adverse events.

Given that CFS was not recorded as an adverse event, it allowed the manufacturers to claim that CFS is not a ‘known adverse event with the vaccine’ and so to discount every case that was reported subsequently.

## Rotavirus vaccine trials

Box 5 describes how adverse events, recorded in a randomized clinical trial (RCT) and sent to the regulatory authority for vaccine approval and license, are not made public. This goes against the European Court of Justice ruling that clinical study reports are made publically accessible.

## Other problems with recording and reporting AEFI

Box 6 describes how the Periodic Safety Update Reports (PSUR) 15 and 16 of Infanrix Hexa and the findings from the reports was opened to public scrutiny by an Italian court. Box 7 describes how PSUR 19 was obtained under the Freedom of Information rules and shows how deaths reported in PSUR 16 were deleted from PSUR 19, when it was evident that the reported deaths exceeded the deaths expected by chance ^22^. In 1986 President Ronald Reagan signed the National Childhood Vaccine Injury Act (NCVIA) (42 U.S.C. §§ 300aa-1 to 300aa-34) which created a no-fault system to compensate vaccine related injuries. This made it difficult to sue vaccine manufactures. It also set up Vaccine Adverse Event Reporting System (VAERS) mandating the reporting of adverse events. Box 8 describes the changes that prevent patients from holding manufacturers to account for adverse events caused by their products. Box 9 shows how AEFI data is no longer available easily. While on the one hand, the new classification discounts AEFI as ‘Not a case of [AEFI]’, safety data is being manipulated and made inaccessible.

**Box 6. Periodic safety update reports : unfit for public consumption?**

Justice Nicola Di Leo in Italy made public the ‘confidential’ 15th and 16th Periodic Safety Update Report (PSUR) on Infanrix hexa (GlaxoSmithKline Biological) and this is now available on the Internet ^a^.

Pages 246-9 document an analysis of the number of ‘sudden deaths’ after receiving the vaccine to examine if it exceeds the number of deaths one could expect from the natural background incidence of sudden death. The background incidence was calculated as 0.454/1000 in the first year and 0.062/1000 live births in the second year. No allowance is made for the notoriously poor AEFI reporting rate. The number of sudden deaths expected to occur by chance between day 1 and 20, is tabulated in Table 36 on page 24. The denominator used to examine deaths following vaccination is the number of doses of the vaccine distributed not the number of children vaccinated. This denominator would dilute any potential signal because many more vaccine doses are distributed than are actually administered!

Further, the number of doses actually administered may be appropriate for milder reactions that can recur with each dose, but it is not appropriate for deaths which can happen only once. Appendix 5A shows that 13 fatal cases were reported. There were more deaths after the first dose than after the second and third doses and the deaths after the second was more than after the third dose. This pattern is commonly seen with AEFIs that are causatively related. The appropriate denominator in all these cases is the number of babies vaccinated.

There were 42 deaths in the first three days after vaccination where there were only 16 deaths in the next 3 days. The fact that the deaths were clustered soon after vaccination suggests that the deaths may be related to the vaccination event.

Patient safety data should not be considered as trade secrets by any stretch of imagination. The practice of keeping safety reports confidential permits such data manipulation in a cosy relationship with the regulators, away from public scrutiny. Such practice ought to be reformed.

**Reference**

a http://autismoevaccini.files.wordpress.com/2012/12/vaccin-dc3a9cc3a8s.pdf Accessed 12/11/15

Box 7. **EMA and Failure of Regulatory Oversight: absence of critical appraisal of PSUR**

GlaxoSmithKline (GSK), 19th confidential periodic safety update reports ^a^ (PSUR 19 (deaths up to October 22, 2014)) on Infanrix hexa makes interesting reading. Infanrix hexa has all the components of the pentavalent vaccine except that it has replaced the whole cell pertussis with an acellular pertussis component and, in addition, it has injectable polio vaccine. The cumulative number of deaths after vaccination reported in the 19th report is less than that reported in the 16th PSUR. It can be seen that deaths in children older than 1 year was significantly higher than the deaths expected by coincidence, if the deaths deleted from the 16th PSUR were restored ^b^.

It appears that the EMA accepts PSUR reports filed by manufacturers without reviewing them critically. Regulatory authorities internationally rely on due diligence by the EMA in such circumstances. This may need to be reappraised.

**Reference**

a. a. http://ijme.in/wp-content/uploads/2017/09/infanrix-pusr.pdf
b. b. http://ijme.in/articles/infanrix-hexa-and-sudden-death-a-review-of-the-periodic-safety-update-reports-submitted-to-the-european-medicines-agency/?galley=html

**Box 8. Product liability: protecting patients not patents.**

Hexavac - a hexavalent vaccine (DTaP-IPV-HepB/Hib) - was withdrawn by the manufacturers without giving reasons after 5 cases of SIDS were reported by Zinka within 48 hours of being administered the vaccine ^a^. vonKries found that in the 2nd year of life, the standardized mortality rate (SMRs) for sudden unexplained deaths (SUD) within 1 day of vaccination was 31.3 (95% CI 3.8–113.1); and within 2 days after vaccination it was 23.5 (95% CI 4.8–68,6) ^b^.

Similarly RotaShield was voluntarily removed from the market after 12 cases of intussusceptions were reported. The background rate of intussusceptions at this age was 5 times the risk of intussusceptions from the vaccine. There was no biologically plausible explanation to link the intussusceptions to the immunization. Yet the vaccine was withdrawn ^c^.

The manufacturers withdrew the vaccines voluntarily without indicating the reasons. It is not clear whether the prospect of product liability suits influenced manufacturer caution.

Two significant changes have taken place after 1980. The threat of vaccine manufacturers being held responsible for marketing a defective product has diminished greatly as a consequence of these changes.

1. A no-fault compensatory mechanism has been put in many countries in the 1980s and 1990s ^d^ This means that vaccine injured children need not provide clear evidence of negligence as cause of the harm, before they qualify for compensation. However, it also means that manufacturers do not have to admit to faults. The risk of product liability has now greatly decreased with no fault compensation being provided by governments. As a result, manufacturers may be emboldened to be more reckless on vaccine safety issues.
2. The second significant change was in 2013, when the methodology for assessment of AEFI was completely overhauled. It is no longer sufficient to show temporal association of the AEFI happening repeatedly. The flow diagram below depicts all conditions that need to be satisfied before an AEFI is labelled ‘Consistent causal association to immunization’. This too could embolden manufacturers to be more reckless with regard to adverse reactions.

**References**

a. Zinka B, Rauch E, Buettner A, Ruëff F, Penning R. Unexplained cases of sudden infant death shortly after hexavalent vaccination. *Vaccine.* 2006;24:5779-80.
b. vonKries R, Toschke AM, Strassburger K, Kundi M, Kalies H, Nennstiel U, Jorch G, Rosenbauer J, Giani G. Sudden and unexpected deaths after the administration of hexavalent vaccines (diphtheria, tetanus, pertussis, poliomyelitis, hepatitis B, Haemophiliusinfluenzae type b): is there a signal? *Eur J Pediatr.* 2005;164:61-9.
c. Center for Disease Control and Prevention. Rotavirus Vaccine (RotaShield) and Intussusception. http://www.cdc.gov/vaccines/vpd-vac/rotavirus/vac-rotashield-historical.htm
d. Looker C, Kelly H. No-fault compensation following adverse events attributed to vaccination: a review of international programmes. Bulletin of the World Health Organization 2011;89:371-378. Available at http://www.who.int/bulletin/volumes/89/5/10-081901/en/ Accessed on 23/10/15

Box 9. **Difficulties in accessing AEFI data**

1. **Polio and Acute Flaccid Paralysis in India**
  As awareness of adverse events is increasing among the public it is becoming more difficult to access data on these adverse events. The National Polio Surveillance provided monthly data on acute flaccid paralysis in India. An analysis of the data showed that in 2011, an additional 47,500 children were newly paralysed in the year, over and above the standard 2/100,000 non-polio AFP that is generally accepted as the norm. The non-polio AFP rate best correlated with the cumulative number of doses received in the previous three years ^a^.
  The analysis was repeated after 2 years when the number of doses administered to children below 5 was reduced and it showed the AFP rate had begun to decline ^b^.
  However, the data is no longer provided on the National Polio Surveillance Project/WHO website.
2. **Data Analysis Prints on Vaccines**
  Medicines and Healthcare products Regulatory Agency (MHRA) of the government of UK provides easily accessible Drug Analysis Prints and interactive Drug Analysis Profiles (iDAPs) ^c^ from ‘Yellow Card’ notifications of adverse events. But this is not provided for vaccines. One is required to request MHRA Pharmacovigilance for this.

**References**

- Vashisht N, Puliyel J. Polio programme: let us declare victory and move on. Indian J Med Ethics. 2012 Apr-Jun;9(2):114-7.
- Vashisht N, Puliyel J, Sreenivas V. Trends in nonpolio acute flaccid paralysis incidence in India 2000 to 2013. Pediatrics. 2015 Feb;135 Suppl 1:S16-7. doi: 10.1542/peds.2014-3330DD. PMID: 26005734

### Biological plausibility: reactions with multi-valent vaccines

Looking at the VAERS data of deaths after immunization, Goldman and colleagues found there was more mortality among babies who had received five to eight vaccines together, compared to those receiving fewer vaccines ^23^. In the case of Boatman v. Secretary of Health and Human Services, 13-611 (Fed. CI 2017) where the infant aged 4 months had received 7 vaccine antigens on one day, the court, after hearing expert opinion, held that vaccine-stimulated inflammatory-cytokines can act as neuro-modulators and cause depression of the serotonergic 5-hydroxytryptophan (5-HT) system in the infant medulla and blunt the normal chemo-sensitive response to excess carbon dioxide and this can result in the death of vulnerable infants during sleep. Multiple vaccines provoke greater release of cytokines. Hill’s criteria of a dose- response gradient (number of antigens in this case), may be satisfied here ^10^.

### Multiple vaccines limited to 5 in the Italian Army

The harm from vaccine-stimulated cytokines is not limited to infancy. The Final Report of the Italian Parliamentary Committee (Doc. XXII-bis N.23) inquiry into cases of death and severe injury affecting Italian personnel assigned to military missions abroad, has recommended that no more than 5 monovalent single-dose vaccines may be given simultaneously to military personnel, in order to avoid adverse reactions. All this suggests the need for caution in using multiple vaccines simultaneously. Ironically, while it is proscribed for healthy adult army men, Hexavac (which combines 6 antigens) is still licensed for use in infants in Italy. 

## Revised AEFI classification and the precautionary principles

It is evident from the discussion earlier that the revised AEFI evaluation scheme produced by the CIOMS / WHO is designed to deny the possibility that any newly observed adverse event may be causally related to the immunization. The AEFI manual states ‘Allegations that vaccines / vaccination cause adverse events must be dealt with rapidly and effectively. Failure to do so can undermine confidence in a vaccine and ultimately have dramatic consequences for immunization coverage…’ ^12^

**Figure 2.**
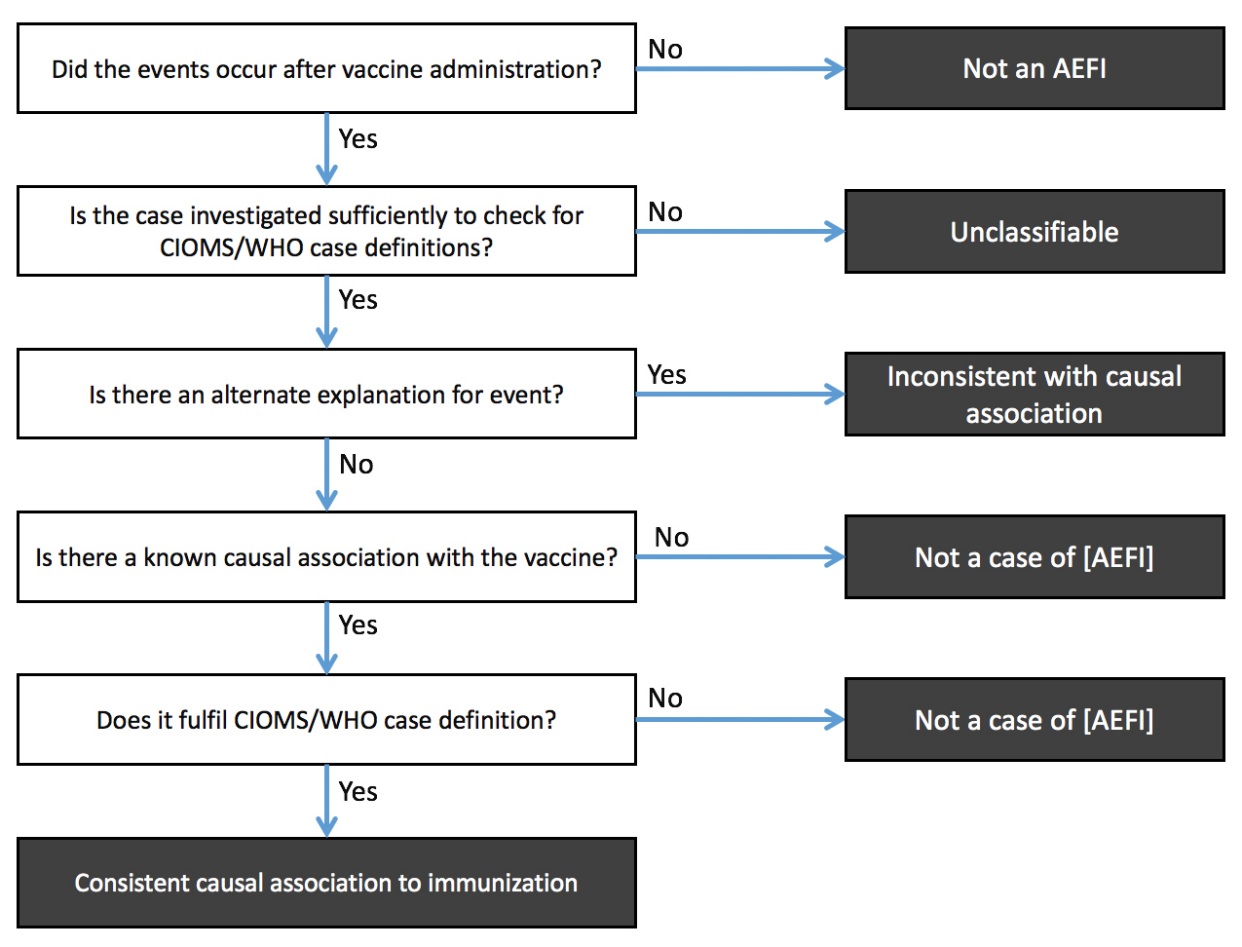
Pathway to achieving ‘consistent causal association to immunization’ status.

Figure 2 shows how all cases AEFI except those that are known adverse effects of vaccine are classified as not causally related.

The AEFI-denialism is a clear violation of the ‘precautionary principle’ ( European Union law), which mandates that ‘when an activity raises threats of harm to the environment or human health, precautionary measures should be taken even if some cause and effect relationships are not fully established scientifically. Society and Government is urged that until the full scientific evidence is available, where there is evidence of risk, it must take precautionary measures’. This new AEFI classification scheme that allows for an outright denial of any new causative association with vaccination could also fall foul of Article 2 European Convention on Human Rights (Art 2 ECHR), which mandates governments to establish a framework of laws, precautions, and means the enforcement of which will, to the greatest extent reasonably practicable, protect life.

Paradoxically, the AEFI algorithm is said to be for vaccine safety. Perhaps we need a scheme for public safety rather than vaccine safety.

The story of pentavalent vaccine was introduced at the beginning of this paper and is summarised in Box 10. It is primarily a vaccine used in developing countries where AEFI surveillance is poor, the press is less vigilant to report adverse events and where drug regulation is less strict. (The richer countries in the West, Europe and the USA, do not use the whole cell pertussis vaccine; so this vaccine is not marketed in those countries.) Isolated cases of unexplained deaths continue to be reported in the press. With the new AEFI classification, in the absence of ‘epidemiological evidence’ linking deaths to the vaccine, these deaths have been passed off as ‘coincidental’ SIDS deaths. Epidemiological evidence, however, is now available linking the deaths to vaccine.

Box 10. **The vaccine that changed the definition of AEFI**

**The story of pentavalent vaccine**

In 1949 the DTP vaccine was introduced ^a^ against diphtheria, tetanus, and pertussis. The first two were frequently fatal diseases. However, DTP was responsible for neurological adverse effects, seizures, encephalopathy, and hypotensive episodes (HHE) ^b^. An acellular DTaP was developed and this has replaced DTP in the West.

In 1981 Hepatitis B was introduced ^a^. Hepatitis B infection can cause chronic liver disease and hepatocellular carcinoma (HCC), especially if acquired at birth. Vaccine uptake was poor in developing countries. One reason was that, although Hepatitis B was common in the potentially large vaccine uptake countries like India, the incidence of HCC was very low ^c^. It is now thought that newborn babies in India may be protected in the early years (where the chance of becoming a chronic carrier is worst) by passive immunity from mother to babies. This may be lost once vaccine use becomes widespread and there could be a paradoxical increase in HCC ^d^.

In 1987 the protein-conjugated Haemophilus influenza type b vaccine was introduced. The incidence of invasive disease with Haemophilus influenza type b in Asia is low ^e^ perhaps due to cross-protection from other bacteria that have cross-reactive antigens to the Hib capsular polysaccharide ^f^. The uptake of Hib vaccine was poor in Asia.

It is said that the Pentavalent vaccine was introduced to improve the uptake of Hib and Hepatitis B, by combining new underused vaccines with a prior UIP vaccine like DTP as a way for the new vaccines to get a piggyback ride into the UIP ^g^. The pentavalent vaccine was used only in developing countries which had not switched to DTaP.

Pentavalent vaccine has been associated with deaths. In the investigation of deaths in Sri Lanka, rather than reporting that the vaccine was ‘probably’ related to the vaccine, the WHO experts deleted the categories ‘probable’ and ‘possible’ from the Brighton classification. This ad-hoc improvisation was reported in medical journals. The AEFI classification was then formally revised so that reactions (deaths in this case) noticed for the first time in Phase 4 trials (post marketing trials) could all be classified as 'Inconsistent with causal association to immunization’ and passed off as ‘coincidental SIDS deaths’.

A new study involving 45 million infants given DTP vaccination and 25 million who received pentavalent vaccine now provides epidemiological evidence that the odds of death after Pentavalent was doubled (OR 1.98 (95% CI 1.65 to 2.38)) compared to DTP. There were 122 additional deaths (95% CI: 101-145) within 72 hours, reported to the government surveillance system, due to the switch from DPT to pentavalent vaccine. A large number of these deaths could have been avoided had the AEFI manual not been revised and the AEFI were evaluated earlier. In fact it is well documented that the combined DTP-Hepatitis B-Hib vaccine causes more local reactions and it is less effective than when they were administered separately ^h^. Protection against these disease could have been better if the vaccines were administered separately.

**References**

a. http://www.immunize.org/timeline/
b. https://www.sciencedirect.com/science/article/pii/0264410X9190283C
c. https://www.ncbi.nlm.nih.gov/pubmed/?term=Hepatitis+B+in+India%3A+Systematic+review+%26+report+of+the+_IMA+sub-committee+on+immunization
d. https://www.ncbi.nlm.nih.gov/pubmed/29318526
e. https://www.ncbi.nlm.nih.gov/pmc/articles/PMC2557750/
f. https://www.ncbi.nlm.nih.gov/pubmed/?term=Natural+immunity+to+Haemophilus+influenza+b+in+infancy+in+Indian+children
g. https://www.researchgate.net/publication/238069905_New_combination_vaccines_Backdoor_entry_into_India%27s_universal_immunization_programme
h. https://www.ncbi.nlm.nih.gov/pubmed/19588375

To examine if deaths following pentavalent vaccine were merely coincidental SIDS deaths, a study of 45 million infants given DTP vaccination and 25 million who received pentavalent vaccine was undertaken. The study assumed that all the deaths (self-reported to the government surveillance system with 72 hours of vaccination) associated with DPT could be coincidental SIDS deaths, but any increase in the death rate after pentavalent vaccine must be assumed to have been caused by pentavalent vaccine. The odds of death after pentavalent vaccine was doubled (OR 1.98 (95% CI 1.65 to 2.38)) compared to DTP. There were 4.7 additional deaths (95% CI: 3.5-5.9) per million vaccinated with Pentavalent vaccine instead of DTP ( *p*<0.0001). By the time this evidence was put together, 122 excess deaths (95% CI: 101-145) had been reported to the government, due to the switch from DPT to pentavalent vaccine. The contribution of the new AEFI classification in this delay in recognizing the problem is stark ^24^.

### The need for revising Brighton

The revised classification have removed the categories ‘probably’ and ‘possible’ from the AEFI classification - very much like the experts who investigated the Sri Lanka deaths. This appears to be motivated by a laudable desire to reduce vaccine hesitancy and the attendant risk of vaccine preventable disease. The Sri Lanka report says, “Cases were classified in this review as unlikely where, in spite of not having evidence that the vaccine(s) contributed to the adverse event or the outcome of death, conclusive evidence regarding an alternate cause (or causes) of the event and outcome was lacking. This meant that we considered that classifying the AEFI in the category ‘unrelated’ was not fully justified (as it could not be conclusively attributed to another cause). In such cases, we go further to state that the conclusion of ‘unlikely’ means that the vaccine is not the major cause of death even in those cases where we discuss the possibility that the vaccine(s) or vaccination may have unmasked an underlying condition”

It seems the Sri Lankan experts were reluctant, even to classify the deaths as ‘unlikely’, as it could be interpreted to mean there was some likelihood of causal association. To quote from the report, “Unlikely: In defining this category, the panel took note of the fact that the WHO category ‘unlikely’ is often interpreted to mean that there is (conversely) some likelihood of a causal association between the adverse event and the vaccine(s) administered.” 

One can speculate that same reasoning and the motivation (to ally public anxiety of a causal association between AEFI and vaccination), would have provided the impetus for the revised AEFI classification.

### The aftermath

That vaccines do more good than harm is taken as an article of faith, a dogma, a tenet. If the purpose of this exercise i AEFI-denialism is to prevent undermining confidence in vaccines, the scheme does not seem to be working. Indeed, public scepticism seems to be increasing rather than diminishing with these efforts at reassurance that vaccines are safe ^25, 26^. Epidemics of vaccine preventable disease have resulted ^27^.

The response in some states in the United States has been to make vaccination mandatory for admission to public schools. Personal and religious belief exemptions for vaccination are not be allowed in California, effective July 1, 2016. The 2016 debates among US Republican Presidential aspirants suggest that there is a lack of widespread support for this measure. The Department of Health and Human Services Office for Civil Rights has now set up the Conscience and Religious Freedom Division to which individuals can complain if their conscience or religious freedom have been abridged. How these forces will interact is anyone’s guess, but the present scenario augur badly for public trust in vaccines and voluntary vaccination.

### Where do we go from here

The AEFI manual needs to be urgently reevaluated and revised. We need to build a better system that picks up problems and at the same time does not create a mistrust of vaccines that have been associated with a major reduction in child mortality.

Adverse reaction and deaths may not show up as significantly increased in small safety studies. However, records of all deaths and serious adverse events following vaccinations should be maintained and periodically reviewed for safety signals. The practice of discarding these records as ‘inconsistent causal association to immunization’ needs to change. Comparisons of the adverse events of vaccines given at the same age, as was done with DTP and pentavalent vaccine, may help to identify adverse events related to one of the vaccines. Sex specific incidence of adverse events may also act as a pointer. Till we develop a better system, it may be advisable to fall back on the time tested WHO-UMC casualty categories and the Brighton categories and to err on the side of child safety.
