# Supplementary material for: Revised World Health Organization (WHO)’s causality assessment of adverse events following immunization—a critique
Source: F1000Res. 2018 May 29;7:243. Originally published 2018 Feb 28. [Version 2] doi: 10.12688/f1000research.13694.2 (PMC6039921; doi:10.12688/f1000research.13694.2)
Supplement: Peer review history

# Peer review: Referee response for version 2

**DOI:** 10.5256/f1000research.15756.r34468

**Published:** 2018-07-05

**Authors:** Peter Aaby

**Affiliations:** 1 Bandim Health Project, Bissau, Guinea-Bissau

I am satisfied with the revision. Many errors and inconsistencies have been corrected. The narrative qualities have improved. The way forward is helpful.

 Including the non-specific effects of vaccines as potential AEFIs is important.

 In general the fact-boxes are easier to understand and helpful now. I did not understand in Box 6 how we came from the 13 fatal cases reported in  Appendix 5A to the 42 deaths said to have occurred in the first 3 days.

I have read this submission. I believe that I have an appropriate level of expertise to confirm that it is of an acceptable scientific standard.

# Peer review: Referee response for version 2

**DOI:** 10.5256/f1000research.15756.r34467

**Published:** 2018-06-04

**Authors:** Tom Jefferson

**Affiliations:** 1 Centre for Evidence Based Medicine, University of Oxford, Oxford, UK

The author's responses seem reasonable to me, although I do not necessarily agree with some of them. As the errors of fact have been corrected, the paper can go ahead as far as I am concerned.

I have read this submission. I believe that I have an appropriate level of expertise to confirm that it is of an acceptable scientific standard.

# Peer review: Referee response for version 1

**DOI:** 10.5256/f1000research.14875.r31300

**Published:** 2018-04-03

**Authors:** Peter Aaby

**Affiliations:** 1 Bandim Health Project, Bissau, Guinea-Bissau

The authors are to be complimented for having conducted this study. Proper handling of AEFIs is very important if we are to maintain trust between public health vaccinology and the community. However, I am missing the authors’ specific suggestions for how to improve the situation. As discussed below there are also details of presentation which could be improved.

 There is a rather detailed description of the changes in the definition of causality in relation to the current concept of AEFI. However, I am missing some presentation of where is the AEFIs concept coming from historically and what is the underlying theoretical biological model of why AEFI might occur and how does that affect how AEFI are observed, reported and used. Furthermore, what are the regulators requirements? Apparently the dominant thinking is that vaccines only induce disease specific memory. Presumably genetic variability may in rare cases affect how this biological process takes place and this could cause specific AEFIs. What else are the causes of other AEFIs: co-incidental infections or chronic disease, co-administration of drugs or other vaccinations? Most of such events can presumably be rejected as not “caused” directly by the vaccine.

 However, the concept of vaccines may be changing. WHO experts have recognized that vaccines may have non-specific effects (NSEs) with consequences for child survival ^1^. Apparently, through epigenetic and metabolic changes, vaccines can reprogram the immune system and upregulate or downregulate both the innate and the adaptive immune system ^2^^-^^5^. If that is the case there is room for both beneficial and deleterious unexpected events following immunization (UEFI). Proper monitoring systems should also be able to detect beneficial UEFIs; for example, we have found that BCG reduces the risk of neonatal sepsis in low-birth weight children ^6^. On the other hand, DTP consistently increases female relative to male mortality, also in societies that have no sex-differential treatment ^7^. This is “unnatural” since there was no excess female mortality in the pre-vaccination era in West Africa ^8^. This being the case there should be room not only for the short-term AEFI as in the current system (14 days?) but also for much more protracted biological processes being classified as AEFI/UEFI. This would require new standards for how UEFI/AEFI are observed and registered.

 Parallel with the description of the changes in the definition of AEFI, there is a series of examples where the authors apparently think there are real differences in mortality/safety issues between different vaccination groups. I have noted at least:  Pentavalent vaccine and congenital heart disease; MMR and autism in African American children; Hexavac; Rotavac; HPV and chronic fatigue syndrome; Pentavalent vaccine vs DTP for SIDS. ****Sometimes these safety issues are mentioned in passing as examples in the discussion of the processes related to AEFI assessment. I found it sometimes unclear whether these example were presented in their own right as safety issues or whether they were only meant to illustrate problems in the assessment of AEFI, e.g. safety reports not forthcoming, etc. Sometimes the presentation was too short or unclear to be really convincing; for example, I had problems with the ROTAVAC story (box 5). It is unclear why it is said in Box 5: “Other rotavirus vaccines that do not reduce incidence of diarrhoea or increase the incidence of diarrhoea instead of decreasing it, have been published (b)”. The paper which is referenced apparently reported a 40% reduction in rota-diarrhoea. If the problem is that overall diarrhea was not reduced I think this can be present more clearly. 

 I think the paper would be stronger/more convincing if the safety-issues that the authors believe have been documented as safety concerns were presented as safety-case stories in specific boxes; the effect of Pentavalent vaccine on SIDS is apparently such a concern. Then the text on the changes in the assessment of AEFI could refer to this or that AEFI problem which was illustrated in the safety-case stories. On the other hand if the story is about mismanagement of the assessment of AEFI, then the cases should be presented as such without implying a causal link between vaccination and AEFI; for example box 3 is an example of poor public communication but it has hardly been documented that MMR causes autism.

**Abstract:**

 It should not be assumed that ”Of course, vaccines that caused deaths in the control-trials stage would not be licensed.” RTS,S malaria vaccine was recently approved by EMA but the trial data indicate that RTS,S compared with control vaccines was associated with 2-fold higher mortality for girls ^9^^,^^10^. Neither the authors nor EMA apparently analysed the mortality data, overall or by sex.

 The example with cardiac failure in children is not presented in the paper and should therefore not appear in the abstract unless it is fully described in the paper. The case might well warrant further presentation in the paper itself.

**Introduction**

 Being presented with the Sri Lanka and Vietnam cases in the first paragraphs, the reader is left wondering what was the implications of the WHO experts’ classifications. Was the pentavalent vaccine (Penta) reintroduced in the countries and how did that decision come about?

**Causality assessment**

 In the long description of changes in the manual for AEFI assessment, it would be good to have an explanation of WHO’s own justification for these changes.

 Sometimes the text appears to have been written some years back but have been maintained unchanged in in the current 2018-version. For example in Box 3 it is said that “Thomson has now been granted whistleblower status by the Obama administration”. By now this sentence should probably be: “Thomson was granted whistleblower status by the Obama administration”. Similar in the conclusion it is said that if the debates among Republican presidential aspirants “are anything to go by”. By now it can no longer be “are”.

 Box 10: this sentence has problems: “In fact combined DTP-Hepatitis B-Hib vaccine causes more there were more local reactions and it is less effective than when they were administered separately.”

 Page 10: Biological plausibility.

 There appears to be an increasing trend to dismiss “unexpected observations”/unpleasant observations with the argument that there is no “biological plausibility”. This was one of the arguments used by WHO experts to dismiss that high-titre measles vaccine (HTMV) could be associated with excess female mortality ^11^. There can obviously not be biological plausibility for a pattern just detected, that no one has ever thought about. The only relevant question is whether a pattern is repeatable – arguments about biological plausibility should not be allowed to dismiss observations of potential AEFIs. The excess female mortality was repeated in subsequent studies and WHO eventually withdrew the HTMV (1992).

 I found this sentence strange: “Slate investigated of the randomised trials of human papillomavirus (HPV) vaccines and found that potential side effects were collected for only two weeks in the year long study.”

 Page 13: “PV” has not been defined as the abbreviation for pentavalent vaccine.

 The comparison of DTP and pentavalent vaccine is frightening. Please indicate whether it is SIDS death or all-cause deaths when it is said for example: “The odds of death after pentavalent vaccine was doubled”. Since it is your study I would have indicated that to the readers: “To examine if deaths following Pentavalent vaccine (PV) were merely coincidental SIDS deaths, we undertook a study of 45 million infants given DPT vaccination and 25 million who received PV”. Given the scary character of this report a bit more information on methods in data collection and analysis would be appropriate. Any hypothesis of why there would be a two-fold difference in SIDS (?) mortality? Did the patterns differ for boys and girls? We have found that DTP and Penta are both associated with much higher female-than-male all-cause mortality rates ^7^^,^^12^.

**Conclusion**

 I do not think the conclusion is really a conclusion to the content of the paper.

 How do we proceed from here? How can we built a better system that finds even the AEFIs we do not want to see and had not expected – and at the same do not create mistrust in the vaccines (BCG, measles vaccines, OPV) which are associated with major reductions in child mortality in low-income countries.  What time-frame should be used? AEFI should always be presented by sex. If there are sex-differential patterns of AEFI it might enhance the credibility of this patterns as a true AEFI since we have found sex-differential effects on mortality of most of common vaccines.

 Biological plausibility should not be used to dismiss any new and unexpected pattern. There is now evidence that vaccines may reprogram both the innate and the adaptive immune system epigenetically with effect on general susceptibility to non-targeted infections ^2^^,^^5^. Hence, the starting point should be that ***unlikely effects are likely*** because we have never examined the possibility.

 It is standard practice in small safety study with deaths to dismiss them because we cannot see a connection. However, deaths following vaccinations should always be classified as potential-even-though-unlikely AEFIs. Otherwise we cannot accumulate the data and detect patterns we had not imagined.  For example, when DTaP was tested in an RCT in Sweden there were 4 deaths among 2847 vaccinated children but none among 954 controls ^13^. Though the authors recognized that 4 deaths was too high and would have been significant if the whole Swedish population of eligible children had been used as controls, the study could find no link between the vaccine and the deaths.  All properly conducted studies from low-income countries have found DTwP to be associated with increased child mortality ^14^^-^^16^.

I have read this submission. I believe that I have an appropriate level of expertise to confirm that it is of an acceptable scientific standard, however I have significant reservations, as outlined above.

## References

[1] HigginsJPSoares-WeiserKLópez-LópezJAKakourouAChaplinKChristensenHMartinNKSterneJAReingoldAL: Association of BCG, DTP, and measles containing vaccines with childhood mortality: systematic review. *BMJ*.2016;355:i5170 2773783410.1136/bmj.i5170PMC5063034

[2] BennCSNeteaMGSelinLKAabyP: A small jab - a big effect: nonspecific immunomodulation by vaccines. *Trends Immunol*.2013;34(9) : 10.1016/j.it.2013.04.004 431-9 10.1016/j.it.2013.04.004 23680130

[3] KleinnijenhuisJQuintinJPreijersFJoostenLAIfrimDCSaeedSJacobsCvan LoenhoutJde JongDStunnenbergHGXavierRJvan der MeerJWvan CrevelRNeteaMG: Bacille Calmette-Guerin induces NOD2-dependent nonspecific protection from reinfection via epigenetic reprogramming of monocytes. *Proc Natl Acad Sci U S A*.2012;109(43) : 10.1073/pnas.1202870109 17537-42 10.1073/pnas.1202870109 22988082PMC3491454

[4] KleinnijenhuisJQuintinJPreijersFBennCSJoostenLAJacobsCvan LoenhoutJXavierRJAabyPvan der MeerJWvan CrevelRNeteaMG: Long-lasting effects of BCG vaccination on both heterologous Th1/Th17 responses and innate trained immunity. *J Innate Immun*.2014;6(2) : 10.1159/000355628 152-8 10.1159/000355628 24192057PMC3944069

[5] ArtsRJWMoorlagSJCFMNovakovicBLiYWangSYOostingMKumarVXavierRJWijmengaCJoostenLABReuskenCBEMBennCSAabyPKoopmansMPStunnenbergHGvan CrevelRNeteaMG: BCG Vaccination Protects against Experimental Viral Infection in Humans through the Induction of Cytokines Associated with Trained Immunity. *Cell Host Microbe*.2018;23(1) : 10.1016/j.chom.2017.12.010 89-100.e5 10.1016/j.chom.2017.12.010 29324233

[6] Biering-SørensenSAabyPLundNMonteiroIJensenKJEriksenHBSchaltz-BuchholzerFJørgensenASPRodriguesAFiskerABBennCS: Early BCG-Denmark and Neonatal Mortality Among Infants Weighing <2500 g: A Randomized Controlled Trial. *Clin Infect Dis*.2017;65(7) : 10.1093/cid/cix525 1183-1190 10.1093/cid/cix525 29579158PMC5849087

[7] AabyPRavnHFiskerABRodriguesABennCS: Is diphtheria-tetanus-pertussis (DTP) associated with increased female mortality? A meta-analysis testing the hypotheses of sex-differential non-specific effects of DTP vaccine. *Trans R Soc Trop Med Hyg*.2016;110(10) : 10.1093/trstmh/trw073 570-581 10.1093/trstmh/trw073 27856947PMC5155548

[8] AabyPMartinsCLGarlyMLRodriguesABennCSWhittleH: The optimal age of measles immunisation in low-income countries: a secondary analysis of the assumptions underlying the current policy. *BMJ Open*.2012;2(4) : 10.1136/bmjopen-2011-000761 10.1136/bmjopen-2011-000761 22815465PMC3401826

[9] KleinSShannFMossWBennCAabyP: RTS,S Malaria Vaccine and Increased Mortality in Girls. *mBio*.2016;7(2) : 10.1128/mBio.00514-16 e00514-16 10.1128/mBio.00514-16 PMC485026727118593

[10] AabyPRodriguesAKofoedPBennC: RTS,S/AS01 malaria vaccine and child mortality. *The Lancet*.2015;386(10005) : 10.1016/S0140-6736(15)00693-5 1735-1736 10.1016/S0140-6736(15)00693-5 26545433

[11] AabyPJensenHSambBCisseBSodemannMJakobsenMPoulsenARodriguesALisseIMSimondonFWhittleH: Differences in female-male mortality after high-titre measles vaccine and association with subsequent vaccination with diphtheria-tetanus-pertussis and inactivated poliovirus: reanalysis of West African studies. *Lancet*.2003;361(9376) : 10.1016/S0140-6736(03)13771-3 2183-8 10.1016/S0140-6736(03)13771-3 12842371

[12] FiskerABiering-SørensenSLundNDjanaQRodriguesAMartinsCBennC: Contrasting female-male mortality ratios after routine vaccinations with pentavalent vaccine versus measles and yellow fever vaccine. A cohort study from urban Guinea-Bissau. *Vaccine*.2016;34(38) : 10.1016/j.vaccine.2016.07.034 4551-4557 10.1016/j.vaccine.2016.07.034 27475473

[13] StorsaeterJOlinPRenemarBLagergårdTNorbergRRomanusVTiruM: Mortality and morbidity from invasive bacterial infections during a clinical trial of acellular pertussis vaccines in Sweden. *Pediatr Infect Dis J*.1988;7(9) :637-45 305085810.1097/00006454-198809000-00008

[14] AabyPRavnHBennCS: The WHO Review of the Possible Nonspecific Effects of Diphtheria-Tetanus-Pertussis Vaccine. *Pediatr Infect Dis J*.2016;35(11) : 10.1097/INF.0000000000001269 1247-1257 10.1097/INF.0000000000001269 27753772

[15] MogensenSWAndersenARodriguesABennCSAabyP: The Introduction of Diphtheria-Tetanus-Pertussis and Oral Polio Vaccine Among Young Infants in an Urban African Community: A Natural Experiment. *EBioMedicine*.2017;17: 10.1016/j.ebiom.2017.01.041 192-198 10.1016/j.ebiom.2017.01.041 28188123PMC5360569

[16] AabyPMogensenSRodriguesABennC: Evidence of Increase in Mortality After the Introduction of Diphtheria–Tetanus–Pertussis Vaccine to Children Aged 6–35 Months in Guinea-Bissau: A Time for Reflection?. *Frontiers in Public Health*.2018;6: 10.3389/fpubh.2018.00079 10.3389/fpubh.2018.00079 29616207PMC5868131

# Response

**Published:** 2018-05-04

**Authors:** Jacob Puliyel

**Affiliations:** St Stephens Hospital, India

Reviewer 2

 Reviewer Professor Peter Aaby

 Reviewer Comment

**Authors’ response**

 The authors are to be complimented for having conducted this study. Proper handling of AEFIs is very important if we are to maintain trust between public health vaccinology and the community.  I am missing the authors’ specific suggestions for how to improve the situation.

**We thank the reviewer for his detailed review and this compliment.**

**We attempt only to critique the revised AEFI classification. Before one makes an effort to improve it, there has to be an acknowledgement of the flaws in the present system.**

**We make no claim to have developed an alternate system of classification. An appropriate body of experts will need to draft it, if there is a consensus on what is flawed with the present system.**

**We have now introduced a new paragraph entitled: “Where do we go from here”. We have suggested that the WHO-UMC causality categories for drug reactions has stood the test of time (and the older Brighton system was adapted from it) may be used till a better system evolves.**

 There is a rather detailed description of the changes in the definition of causality in relation to the current concept of AEFI. However, I am missing some presentation of where is the AEFIs concept coming from historically and what is the underlying theoretical biological model of why AEFI might occur and how does that affect how AEFI are observed, reported and used. Furthermore, what are the regulators requirements? Apparently the dominant thinking is that vaccines only induce disease specific memory. Presumably genetic variability may in rare cases affect how this biological process takes place and this could cause specific AEFIs. What else are the causes of other AEFIs: co-incidental infections or chronic disease, co-administration of drugs or other vaccinations? Most of such events can presumably be rejected as not “caused” directly by the vaccine.

**We have dealt very briefly with the historic and theoretical background – but within the 8000 word-limit, we could not deal with this in greater detail.**

**Regarding: ‘** **AEFIs concept coming from historically’**

**The other reviewer Prof. Tom Jefferson (TJ) has suggested we introduce the contributions made by the AB Hill and we have made reference to that.**

**We refer to genetic predisposition to AEFI in our article and also how underlying disease like congenital heart lesions can precipitate an AEFI. This paragraph has been added in the main body on the text.**

**In the new version we make reference to non-specific effects of vaccine and we thank the reviewer for his suggestion.**

 However, the concept of vaccines may be changing. WHO experts have recognized that vaccines may have non-specific effects (NSEs) with consequences for child survival ^1^. Apparently, through epigenetic and metabolic changes, vaccines can reprogram the immune system and upregulate or downregulate both the innate and the adaptive immune system ^2^ ^-^ ^5^. If that is the case there is room for both beneficial and deleterious unexpected events following immunization (UEFI). Proper monitoring systems should also be able to detect beneficial UEFIs; for example, we have found that BCG reduces the risk of neonatal sepsis in low-birth weight children ^6^. On the other hand, DTP consistently increases female relative to male mortality, also in societies that have no sex-differential treatment ^7^. This is “unnatural” since there was no excess female mortality in the pre-vaccination era in West Africa ^8^. This being the case there should be room not only for the short-term AEFI as in the current system (14 days?) but also for much more protracted biological processes being classified as AEFI/UEFI. This would require new standards for how UEFI/AEFI are observed and registered.

**We thank the reviewer  for the references.  In our new submission we refer briefly to these NSEs and the benefits and harms than can result.**

 Parallel with the description of the changes in the definition of AEFI, there is a series of examples where the authors apparently think there are real differences in mortality/safety issues between different vaccination groups. I have noted at least:  Pentavalent vaccine and congenital heart disease; MMR and autism in African American children; Hexavac; Rotavac; HPV and chronic fatigue syndrome; Pentavalent vaccine vs DTP for SIDS. Sometimes these safety issues are mentioned in passing as examples in the discussion of the processes related to AEFI assessment. I found it sometimes unclear whether these example were presented in their own right as safety issues or whether they were only meant to illustrate problems in the assessment of AEFI, e.g. safety reports not forthcoming, etc. Sometimes the presentation was too short or unclear to be really convincing; for example, I had problems with the ROTAVAC story (box 5). It is unclear why it is said in Box 5: “Other rotavirus vaccines that do not reduce incidence of diarrhoea or increase the incidence of diarrhoea instead of decreasing it, have been published (b)”. The paper which is referenced apparently reported a 40% reduction in rota-diarrhoea. If the problem is that overall diarrhea was not reduced I think this can be present more clearly. 

 I think the paper would be stronger/more convincing if the safety-issues that the authors believe have been documented as safety concerns were presented as safety-case stories in specific boxes; the effect of Pentavalent vaccine on SIDS is apparently such a concern. Then the text on the changes in the assessment of AEFI could refer to this or that AEFI problem which was illustrated in the safety-case stories. On the other hand if the story is about mismanagement of the assessment of AEFI, then the cases should be presented as such without implying a causal link between vaccination and AEFI; for example box 3 is an example of poor public communication but it has hardly been documented that MMR causes autism.

**They are presented as potential safety problems that seem to get glossed over, by the Revised AEFI assessment methodology. (Please also see next point in row below).**

**Regarding Rotavac the problem is that overall diarrhea is not decreased and this is clarified in the revised text**

**Safety-case stories with Pentavac and Hexavac have been identified as such in the revised manuscript**

**The MMR story Box 3 was about increased autism seen in African American boys vaccinated prior to age of 2 years (compared to those vaccinated after 2 years).**

**Post-hoc, data of many African American children were excluded on the grounds that they did not possess a valid birth certificate –and reanalysis of this truncated data was published to suggest that MMR was not related autism in any group.**

**This suggests a possible link between Autism and MMR (albeit in one specific ethnic and age group).**

   

**In summary, all the examples noted by the reviewer in the article ( Pentavalent vaccine and congenital heart disease; MMR and autism in African American children; Hexavac; Rotavac; HPV and chronic fatigue syndrome; Pentavalent vaccine vs DTP for SIDS) are AEFI that are ‘probably’ related causatively with vaccination which are not acknowledged.**

**In the case of MMR and Hexavac the new revised classification cannot be blamed as the data itself was falsified (Many African American boys excluded from MMR study and deaths deleted from PSUR 19).**

**Abstract**

 It should not be assumed that ”Of course, vaccines that caused deaths in the control-trials stage would not be licensed.” RTS,S malaria vaccine was recently approved by EMA but the trial data indicate that RTS,S compared with control vaccines was associated with 2-fold higher mortality for girls ^9^ ^,^ ^10^. Neither the authors nor EMA apparently analysed the mortality data, overall or by sex.

 The example with cardiac failure in children is not presented in the paper and should therefore not appear in the abstract unless it is fully described in the paper. The case might well warrant further presentation in the paper itself.

**This sentence  has been corrected in abstract**

**The case of heart failure in children is now included in the paper as suggested by the reviewer** **.**

**Introduction**

 Being presented with the Sri Lanka and Vietnam cases in the first paragraphs, the reader is left wondering what was the implications of the WHO experts’ classifications. Was the pentavalent vaccine (Penta) reintroduced in the countries and how did that decision come about?

**The vaccines were reintroduced after the ‘WHO experts’ report, and this is mentioned now in the revised manuscript.**

**The inevitable follow-up question then is: Were there deaths after it was reintroduced?**

**We know from the data from India that using the Revised AEFI manual, each death is certified as ‘inconsistent with causal association’ on the grounds that death has so far never been acknowledged as having occurred in epidemiological studies with the vaccine. This is explained in the paper with reference from literature.**

**Causality assessment**

 1. In the long description of changes in the manual for AEFI assessment, it would be good to have an explanation of WHO’s own justification for these changes.

 2. Sometimes the text appears to have been written some years back but have been maintained unchanged in the current 2018-version. For example in Box 3 it is said that “Thomson has now been granted whistleblower status by the Obama administration”. By now this sentence should probably be: “Thomson was granted whistleblower status by the Obama administration”. Similar in the conclusion it is said that if the debates among Republican presidential aspirants “are anything to go by”. By now it can no longer be “are”.

 Box 10: this sentence has problems: “In fact combined DTP-Hepatitis B-Hib vaccine causes more there were more local reactions and it is less effective than when they were administered separately.”

 3. Page 10: Biological plausibility.

 There appears to be an increasing trend to dismiss “unexpected observations”/unpleasant observations with the argument that there is no “biological plausibility”. This was one of the arguments used by WHO experts to dismiss that high-titre measles vaccine (HTMV) could be associated with excess female mortality ^11^. There can obviously not be biological plausibility for a pattern just detected, that no one has ever thought about. The only relevant question is whether a pattern is repeatable – arguments about biological plausibility should not be allowed to dismiss observations of potential AEFIs. The excess female mortality was repeated in subsequent studies and WHO eventually withdrew the HTMV (1992).

 4. I found this sentence strange: “Slate investigated of the randomised trials of human papillomavirus (HPV) vaccines and found that potential side effects were collected for only two weeks in the year long study.”

 5. Page 13: “PV” has not been defined as the abbreviation for pentavalent vaccine.

 6. The comparison of DTP and pentavalent vaccine is frightening. Please indicate whether it is SIDS death or all-cause deaths when it is said for example: “The odds of death after pentavalent vaccine was doubled”. Since it is your study I would have indicated that to the readers: “To examine if deaths following Pentavalent vaccine (PV) were merely coincidental SIDS deaths, we undertook a study of 45 million infants given DPT vaccination and 25 million who received PV”. Given the scary character of this report a bit more information on methods in data collection and analysis would be appropriate. Any hypothesis of why there would be a two-fold difference in SIDS (?) mortality? Did the patterns differ for boys and girls? We have found that DTP and Penta are both associated with much higher female-than-male all-cause mortality rates ^7^ ^,^ ^12^.

**1. This has not been justified as far as we know. The rationale for revising the Brighton classification has also not been stated explicitly.**

**Reviewer TJ suggested that WHO must be given an opportunity to defend the changes.**

**David Legge and I had written to WHO, before we published the short critique in BMJ referenced in the paper (Reference 14). This is copied in the response to TJ.  There was no response from WHO.**

**This present paper was also sent to WHO after it appeared in F1000 to seek their comments. There has not been any response so far.**

**2. The reviewer is correct that the article has been written in parts and the tense needs to be corrected for consistency. This has now been done.**

**3. We thank the reviewer for this example that has been included in the text.**

**4. The Slate story: that was the point of the Slate report – that adverse events were not recorded properly.**

**5. Abbreviation PV has been removed**

**6. The DPT Pentavalent story is about deaths within 72 hours of vaccination. As babies taken for vaccination are usually not unwell, these must be considered as SIDS deaths in ‘well children’ and comparisons can only be made with the acceptable death rate for ‘well children’ and it must NOT be compared to the ‘all cause death rate’ which includes in the cohort well and unwell children. This is sometimes referred to as the ‘healthy vaccinee effect’**

**When we report** **“The odds of death after pentavalent vaccine was doubled” we DO NOT have to make any extra allowance for the ‘healthy vaccinee effect’ as both DPT and Pentavalent vaccine are given to healthy children. The deaths among children getting pentavalent vaccine was twice as high as those getting DPT**

**There is no hypothesis for the deaths - except as explained in the Boatman case -  that the use of multiple vaccines release more inflammatory  cytokines (than when single vaccines given) which can act  as neuro-modulators and can cause depression of the serotonergic 5-hydroxytryptophan (5-HT) system in the infant medulla and blunt the normal chemo-sensitive response to excess carbon dioxide and this can result in the death of vulnerable infants during sleep.**

**The method of data collection is described in great detail in the reference (which would be too long to reproduce in this paper). It may suffice to say that both DPT and Pentavalent deaths were captured in the same ‘improved’ government surveillance system and the data has been made freely available on-line for rechecking by stake holders, and more studies by future researchers.**

**The records did not specify sex of child.**

**Conclusion**

1. I do not think the conclusion is really a conclusion to the content of the paper.
  How do we proceed from here? How can we built a better system that finds even the AEFIs we do not want to see and had not expected – and at the same do not create mistrust in the vaccines (BCG, measles vaccines, OPV) which are associated with major reductions in child mortality in low-income countries.  What time-frame should be used? AEFI should always be presented by sex. If there are sex-differential patterns of AEFI it might enhance the credibility of this patterns as a true AEFI since we have found sex-differential effects on mortality of most of common vaccines.
  Biological plausibility should not be used to dismiss any new and unexpected pattern. There is now evidence that vaccines may reprogram both the innate and the adaptive immune system epigenetically with effect on general susceptibility to non-targeted infections ^2^ ^,^ ^5^. Hence, the starting point should be that ***unlikely effects are likely*** because we have never examined the possibility.
  It is standard practice in small safety study with deaths to dismiss them because we cannot see a connection. However, deaths following vaccinations should always be classified as potential-even-though-unlikely AEFIs. Otherwise we cannot accumulate the data and detect patterns we had not imagined.  For example, when DTaP was tested in an RCT in Sweden there were 4 deaths among 2847 vaccinated children but none among 954 controls ^13^. Though the authors recognized that 4 deaths was too high and would have been significant if the whole Swedish population of eligible children had been used as controls, the study could find no link between the vaccine and the deaths.  All properly conducted studies from low-income countries have found DTwP to be associated with increased child mortality ^14^ ^-^ ^16^.

 

1. **The conclusion has been revised as suggested**
2. **We have included a paragraph on “Where do we go from here”.**

# Peer review: Referee response for version 1

**DOI:** 10.5256/f1000research.14875.r31301

**Published:** 2018-03-14

**Authors:** Tom Jefferson

**Affiliations:** 1 Centre for Evidence Based Medicine, University of Oxford, Oxford, UK

Thank you for asking my views on this paper.

 This is a very long and detailed examination of the philosophical, historical rationale and principles of causality assessment of possible vaccine harms, chiefly death. This is mixed with the narrative of the changes made by WHO to their own assessment rules.

 The authors use several important examples to make their points.

 I regard this topic as extremely interesting and important and the authors should be congratulated for attempting to pull the main strands together, from David Hume to the Brighton Collaboration.

 Despite my interest I found the manuscript extremely heavy going with a difficult-to-follow thread.

 It soon became apparent that the authors think there has been something akin to an international conspiracy to bury the dead by changing the definitions of probable and likely causality. That may be so, but I could not find any convincing evidence in the paper.

 Here and there inaccuracies and typos add to the distractions. For example in box 10, DTP becomes DPT or the suggestions that Rotashield was withdrawn in 1999 as a consequence of the Brighton criteria. As far as I remember in 1999 we were setting up and had not produced the criteria or any other output yet.

 I would also check the data of Rotashield withdrawal from the market.

 What follows are a few suggestions to improve the manuscript (ms).

 First I would split the ms into 2 parts. One discussing the philosophical-historical basis for causality assessment perhaps as far as Brighton and the second one looking at the more recent changes.

 Here I have two further suggestions to offer.

 Bradford Hill's criteria should be cited, even though they are not a perfect solution as Hill himself recognised. They should be cited because they have had an enormous influence on modern epidemiology (see Geoffrey Rose's variant for example) and because the formulation of some of them (temporality, strength, gradient) is very apt for vaccine exposure. Take temporality for example. The term AEFI which is so extensively cited concedes temporality when in fact temporality is only as good as the vaccination records. Often "AEFI" is used when we are not sure that exposure has taken place at all or that it preceded the clinical event/possible harm. So a balanced discussion of temporality (one of the absolute conditions for determining causality) must include absolute certainty or high probability that exposure preceded the event and that it had taken place at all.  

 Second I would offer the connection of probabilism and Fisherian theory with Hume's problem of induction. I see Fisher's work as the patch that allows us to go on with at least a partially clear conscience, as I do not think there is a solution to Hume's problem as nature is not (and never will be) universally uniform.

 I would tone down the plot theory rhetoric and would seek a written explanation from WHO for their actions. WHO do not have a good track record of answering researchers but the effort must be made and reported. Ditto for any other point which was unclear to the authors. I am not a great believer in plots, blunders fit the picture and my experience better, but the authors must try and get to the bottom of the rationale for the changes and, while at it, they might just want to ask WHO, CIOMS etc. to check the authors' facts and dates (but not their opinions of course).

 Last but not least please ask Brighton whether they were aware of WHO's actions (they must be) and what their views are.

 I hope these suggestions are useful to the authors.

I have read this submission. I believe that I have an appropriate level of expertise to confirm that it is of an acceptable scientific standard, however I have significant reservations, as outlined above.

# Response

**Published:** 2018-05-04

**Authors:** Jacob Puliyel

**Affiliations:** St Stephens Hospital, India

Reviewer 1

Referee Prof Tom Jefferson

Reviewer Comment

**Authors’ response**

This is a very long and detailed examination of the philosophical, historical rationale and principles of causality assessment of possible vaccine harms, chiefly death. This is mixed with the narrative of the changes made by WHO to their own assessment rules.

The authors use several important examples to make their points.

I regard this topic as extremely interesting and important and the authors should be congratulated for attempting to pull the main strands together, from David Hume to the Brighton Collaboration.

**Thanks**

Despite my interest I found the manuscript extremely heavy going with a difficult-to-follow thread.

It soon became apparent that the authors think there has been something akin to an international conspiracy to bury the dead by changing the definitions of probable and likely causality. That may be so, but I could not find any convincing evidence in the paper.

**‘Conspiracy theory’ according to Barkun is a closed and unfalsifiable system and it is merely a matter of faith with no proof.**

**We hope we have critiqued various provisions in the revised manual for AEFI and shown how it can lead to harm – how it can result in delays in the acknowledgment of the problems that can result from vaccines. We hope this is a critique of the revised manual ‘not unfalsifiable conspiracy theory’.**

****

**According to the revised AEFI classification reactions must be ‘known to be associated with the vaccine’ before it is acknowledged as caused by the vaccine. We have shown how new signals can be (and are being) ignored on account of this proviso.**

****

**We merely suggest there is potential for harm inherent in the revised system.**

****

**Regarding the observation**

**“international conspiracy to bury the dead by changing the definitions of probable and likely causality. That may be so, but I could not find any convincing evidence in the paper.”**

**The WHO experts in Sri Lanka removed these categories (‘probable’ and ‘possible’) before they reported the deaths were ‘unlikely’ to be caused by vaccine. This is a verifiable fact. Had the categories not been removed, they would have had to report that 3 death (for with there was no alternate explanation) were ’probably’ related to vaccination.**

****

**Chronologically at least, the new ‘Revised AEFI’ categories  were developed after the Sri Lanka report was criticized in the BMJ etc.**

****

**After the AEFI classification was revised, experts no longer have the mortification of having to report they have deleted ‘Probable’ and ‘possible’. The revised AEFI have eliminated the categories ‘probable/likely’ and ‘possible’.**

****

**In fact the Sri Lanka experts were very keen to absolve the vaccine. They write they felt reluctant to even classify the deaths as ‘unlikely’ to be related to vaccine. I quote from the Sri Lanka  report:**

**“Unlikely: In defining this category, the panel took note of the fact that the WHO category ‘unlikely’ is often interpreted to mean that there is (conversely) some likelihood of a causal association between the adverse event and the vaccine(s) administered….” (The full report is uploaded here for easy access http://www.jacob.puliyel.com/download.php?id=213)**

****

**I have no doubt that the experts are motivated by a laudable desire to reduce vaccine hesitancy and the attendant risk of vaccine preventable disease.**

****

**The reasoning for the revised AEFI categories must be similar to that of the experts of the Sri Lanka report.**

****

**We have added a new paragraph explaining that we feel the motivation for the change was a laudable desire to reduce vaccine hesitancy.**

****

Here and there inaccuracies and typos add to the distractions. For example in box 10, DTP becomes DPT or the suggestions that Rotashield was withdrawn in 1999 as a consequence of the Brighton criteria. As far as I remember in 1999 we were setting up and had not produced the criteria or any other output yet.

I would also check the data of Rotashield withdrawal from the market.

**The errors have been corrected in the revised manuscript.**

****

**The point made by the reviewer about Rotashield is correct. It has been revised. As pointed out by the reviewer, RotaShield was marketed before Brighton was developed. The WHO/UMC system was in place at that time.**

**Corrections have been made in the revised version.**

**Bradford Hill's criteria should be cited, even though they are not a perfect solution as Hill himself recognised. They should be cited because they have had an enormous influence on modern epidemiology (see Geoffrey Rose's variant for example) and because the formulation of some of them (temporality, strength, gradient) is very apt for vaccine exposure. Take temporality for example. The term AEFI which is so extensively cited concedes temporality when in fact temporality is only as good as the vaccination records. Often "AEFI" is used when we are not sure that exposure has taken place at all or that it preceded the clinical event/possible harm. So a balanced discussion of temporality (one of the absolute conditions for determining causality) must include absolute certainty or high probability that exposure preceded the event and that it had taken place at all.**

**Bradford Hill's criteria have been cited. Thanks.**

Second I would offer the connection of probabilism and Fisherian theory with Hume's problem of induction. I see Fisher's work as the patch that allows us to go on with at least a partially clear conscience, as I do not think there is a solution to Hume's problem as nature is not (and never will be) universally uniform.

**We did not take the reviewers suggestion to introduce  Fisherian theory  - to avoid making the  write-up even more complicated. As the reviewer stated: the manuscript is already “extremely heavy going”**

I would tone down the plot theory rhetoric and would seek a written explanation from WHO for their actions. WHO do not have a good track record of answering researchers but the effort must be made and reported. Ditto for any other point which was unclear to the authors. I am not a great believer in plots, blunders fit the picture and my experience better, but the authors must try and get to the bottom of the rationale for the changes and, while at it, they might just want to ask WHO, CIOMS etc. to check the authors' facts and dates (but not their opinions of course).

**The revised manuscript  states explicitly that the motivation for the changes is probably to reduce vaccine hesitancy.**

****

**The WHO was contacted a year prior to publishing this article in the BMJ but there was no response  ( *BMJ* 2017;357:j2449, published 19 May 2017). They were again contacted after the first version of this manuscript was published on F1000Research, so they could respond in the comments section. There is no response so far (as of 12/4/18).**

****

Last but not least please ask Brighton whether they were aware of WHO's actions (they must be) and what their views are.

**Apparently the present Brighton team approves of these changes.  They are now tasked with the responsibility to develop ‘case definition’ for ‘known AEFI’ (See CIOMS/WHO report)**

****
